# Niche-specific metabolic adaptation in biotrophic and necrotrophic oomycetes is manifested in differential use of nutrients, variation in gene content, and enzyme evolution

**DOI:** 10.1371/journal.ppat.1007729

**Published:** 2019-04-19

**Authors:** Audrey M. V. Ah-Fong, Meenakshi S. Kagda, Melania Abrahamian, Howard S. Judelson

**Affiliations:** Department of Microbiology and Plant Pathology, University of California, Riverside, United States of America; Nanjing Agricultural University, CHINA

## Abstract

The use of host nutrients to support pathogen growth is central to disease. We addressed the relationship between metabolism and trophic behavior by comparing metabolic gene expression during potato tuber colonization by two oomycetes, the hemibiotroph *Phytophthora infestans* and the necrotroph *Pythium ultimum*. Genes for several pathways including amino acid, nucleotide, and cofactor biosynthesis were expressed more by *Ph*. *infestans* during its biotrophic stage compared to *Py*. *ultimum*. In contrast, *Py*. *ultimum* had higher expression of genes for metabolizing compounds that are normally sequestered within plant cells but released to the pathogen upon plant cell lysis, such as starch and triacylglycerides. The transcription pattern of metabolic genes in *Ph*. *infestans* during late infection became more like that of *Py*. *ultimum*, consistent with the former's transition to necrotrophy. Interspecific variation in metabolic gene content was limited but included the presence of γ-amylase only in *Py*. *ultimum*. The pathogens were also found to employ strikingly distinct strategies for using nitrate. Measurements of mRNA, ^15^N labeling studies, enzyme assays, and immunoblotting indicated that the assimilation pathway in *Ph*. *infestans* was nitrate-insensitive but induced during amino acid and ammonium starvation. In contrast, the pathway was nitrate-induced but not amino acid-repressed in *Py*. *ultimum*. The lack of amino acid repression in *Py*. *ultimum* appears due to the absence of a transcription factor common to fungi and *Phytophthora* that acts as a nitrogen metabolite repressor. Evidence for functional diversification in nitrate reductase protein was also observed. Its temperature optimum was adapted to each organism's growth range, and its K_m_ was much lower in *Py*. *ultimum*. In summary, we observed divergence in patterns of gene expression, gene content, and enzyme function which contribute to the fitness of each species in its niche.

## Author summary

A key feature of disease is the pathogen's consumption of host metabolites to support its growth and multiplication. Understanding how host nutrients are used by pathogens may lead to strategies for limiting disease, for example by developing inhibitors of metabolic pathways needed for pathogen growth. Feeding strategies of plant pathogens range between two extremes: necrotrophs kill host cells and consume the released nutrients, while biotrophs do not injure host cells but instead acquire nutrients from extracellular spaces in the plant. In this study, a comparison was made between the metabolism of *Phytophthora infestans* (the infamous Irish Famine pathogen) and *Pythium ultimum* during potato tuber colonization. These microbes have close evolutionary histories, but while *Py*. *ultimum* is a necrotroph, *Ph*. *infestans* is a biotroph for most of the disease cycle. It was discovered that distinct patterns of metabolic gene expression, gene content, and enzyme behavior underlie these lifestyles. For example, genes for utilizing compounds that are normally stored within plant cells were expressed more by *Py*. *ultimum*, while *Ph*. *infestans* appeared to synthesize more biosubstances from precursors. Several differences in carbon and nitrogen metabolism were linked to variation in enzyme content and gene expression regulators in the two species.

## Introduction

The most fundamental characteristic of microbial pathogenesis is the use of host nutrients to support pathogen growth [1, 2]. Trophic lifestyles of plant pathogens have been categorized as biotrophic when the host stays alive during the nutrient exchange, or necrotrophic when the pathogen kills and feeds on the remains of plant cells [3]. An additional level of diversity in phytopathogens is that while some are exclusively plant colonizers, others cycle between saprophytism and parasitism. Much has been learned about the tactics that plant pathogens employ to suppress host defenses to maintain biotrophy or kill cells during necrotrophy [3, 4]. However, little is known about how the life strategies of pathogens are reflected in their metabolism.

Pathogens can be considered as occupants of specific environmental niches to which their metabolism has been tailored. Studies of natural and lab-induced variation have demonstrated that metabolism can evolve through multiple processes. Some involve major events such as the loss of a gene, or the acquisition of new functions by lateral transfer [5–7]. Smaller changes include mutations that modify regulatory proteins, alter promoter activity, or change the substrate affinity, reaction kinetics, or allosteric regulation of an enzyme [8–11]. Such mutations occur spontaneously within pathogen populations and when selected may help tune metabolism to a lifestyle, environment, or new host.

*Phytophthora infestans* and *Pythium ultimum* present interesting contrasts in pathogen lifestyles. These species belong to sister genera in the peronosporalean lineage of oomycetes, which cause blights and rots of thousands of important plants [12]. *Ph*. *infestans* triggered the notorious Irish Famine and persists as arguably the most destructive pathogen of potato, infecting foliage and tubers [13]. *Ph*. *infestans* grows biotrophically for most of the disease cycle, producing haustoria and effectors that suppress host defenses and feeding on apoplastic nutrients [3]. *Ph*. *infestans* is classified as a hemibiotroph since host necrosis occurs near the end of the disease cycle [13, 14], although data that indicate that this stage is necrotrophic and not simply necrogenic are sparse. *Py*. *ultimum* also infects potato tubers but is a necrotroph. While tubers colonized by *Ph*. *infestans* remain firm unless secondary pathogens are present, *Py*. *ultimum* transforms tubers into watery rotted tissue while feeding off the liberated nutrients [15]. Like other members of its genus, *Py*. *ultimum* also grows in soil or plant debris [16]. In contrast, most members of the genus *Phytophthora* including *Ph*. *infestans* can only survive in nature by growing on living plants.

The goal of this study was to understand how the metabolism of *Ph*. *infestans* and *Py*. *ultimum* is adapted to their respective lifestyles. Most prior comparisons of pathogens with distinct trophisms have examined infections of different plant species, which complicates analysis since each host may provide dissimilar nutrients [17, 18]. This can be avoided with *Ph*. *infestans* and *Py*. *ultimum* since both infect potato tubers. In an earlier study, we focused on effectors and other pathogenicity factors expressed by these oomycetes during tuber infection [19]. In this paper, we concentrate on metabolism. We annotated metabolic genes of the two species, compared RNA-seq and metabolomic data from tubers and media at different stages of infection, and identified genus-specific differences in metabolic gene content and expression pattern. We also observed shifts in metabolic gene expression during tuber colonization by *Ph*. *infestans* that provide evidence of necrotrophy during the late stages of its disease cycle. We also focused on nitrate assimilation, which appeared to be more active in *Py*. *ultimum*. Measurements of RNA and protein levels, enzyme assays, and isotopic labeling revealed dramatic differences in how the two species use nitrate. Like many organisms, *Ph*. *infestans* prefers to obtain nitrogen from amino acids, using nitrate as a last resort. In contrast, *Py*. *ultimum* assimilates nitrate at all stages of growth regardless of the availability of more economical nitrogen sources. While the assimilation genes in *Py*. *ultimum* were nitrate-induced, this was not the case for *Ph*. *infestans*. How variation in expression pattern and enzyme function relates to each organism's lifestyle is discussed.

## Results

### Annotation of metabolic genes

**Fig 1:**
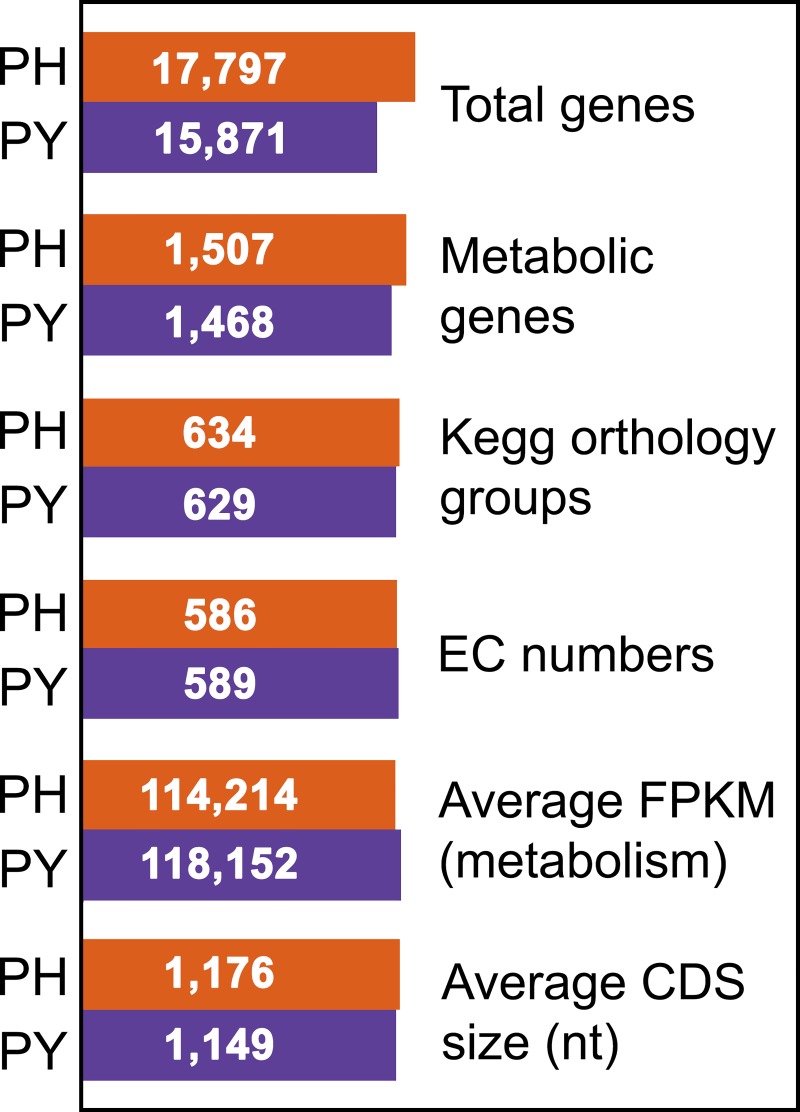
Comparison of metabolic transcriptomes. Indicated for *Ph*. *infestans* (PH) and *Py*. *ultimum* (PY) are the numbers of predicted total genes and those encoding metabolic enzymes. The latter excludes those involved in protein, DNA, or cell wall modification. Also shown for the metabolic genes are the number of KEGG orthology groups, their aggregate expression in RNA-seq studies of hyphae (expressed as counts per million, CPM), and the average size of the transcripts.

Genes from *Ph*. *infestans* and *Py*. *ultimum* were annotated as described in Materials and Methods. This involved primarily the use of the KEGG Automatic Annotation Server (KAAS; [20]), the KEGG Orthology And Links Annotation tool (KOALA; [21]), and protein domain databases. Orthologs between *Ph*. *infestans* and *Py*. *ultimum* were identified using OrthoMCL, and their annotations compared to identify and resolve ambiguities. The genes were then assigned Enzyme Commission (EC) and KEGG ortholog (KO) group numbers, and categorized into pathways using the KEGG schema. Enzymes that synthesize or modify proteins, nucleic acids, and the cell wall were excluded from analysis. We identified 1507 metabolic genes representing 586 EC numbers from *Ph*. *infestans* and 1468 genes representing 589 EC numbers from *Py*. *ultimum* (Fig 1; S1 Table). These represent 8.5% and 9.2% of total genes, respectively. Much of the interspecific differences in gene numbers were due to gene family expansions.

Since our goal was to use RNA-seq data to compare the expression of metabolic pathways in the two species, we performed several preliminary analyses to help predict whether that strategy would be valid. One concern was that differences in gene model quality could lead to false conclusions; for example, if gene models were consistently truncated in one species, this might lead to the inference that its genes were transcribed more weakly. However, such issues did not appear to be significant. The average coding sequence sizes in *Ph*. *infestans* and *Py*. *ultimum* were nearly identical at 1176 and 1149-nt, respectively (Fig 1). Moreover, the aggregate FPKM value (fragments per kilobase per million mapped reads) of metabolic genes during growth in rye media and tubers were very similar, averaging 114,214 and 118,152 in *Ph*. *infestans* and *Py*. *ultimum*, respectively.

### Genus-specific metabolic activities

Several enzymes appeared to lack orthologs in one of the two oomycetes. Many enzymes initially appeared to be species-specific but were later detected as unannotated genes in the assembly. Excluding those, we detected in *Ph*. *infestans* but not *Py*. *ultimum* genes predicted to encode pyruvate phosphate dikinase (*e*.*g*. PITG_03721; EC 2.7.9.1), sorbitol/iditol dehydrogenase (*e*.*g*. PITG_04121; EC 1.1.1.14), salicylate dehydrogenase (PITG_17117; EC 1.14.13.1), and formate dehydrogenase (PITG_13448; EC 1.2.1.2). Based on searching the Fungidb.org and Eumicrobedb.org web sites [22, 23], orthologs of these genes were present in each of eight *Phytophthora* spp. in those databases but not any of the six sequenced members of *Pythium*. We also detected orthologs in *Phytopythium vexans*, which bridges the two genera [24].

The enzymes specific to *Phytophthora* were not distributed widely in other oomycetes. For example, absent from the downy mildews *Hyaloperonospora arabidopsidis* and *Plasmopara halstedii*, two *Albugo* white rusts, two *Aphanomyces* spp., and two *Saprolegnia* spp. were sequences for pyruvate phosphate dikinase (EC 2.7.9.1), formate dehydrogenase (EC 1.2.1.2), and the sorbitol/iditol dehydrogenase (EC 1.1.1.14). The putative salicylate dehydrogenase (EC 1.2.1.65) also appeared to be specific to *Phytophthora*.

Several activities were also predicted to occur in *Py*. *ultimum* but not *Ph*. *infestans*. These included three enzymes involved in thiamine biosynthesis, which has been reported to be absent from haustoria-forming oomycetes [25]. Also lacking from all sequenced *Phytophthora* spp. were glucosamine-6-phosphate deaminase (PYU1_G006886, EC 3.5.99.6) and N-acetylglucosamine-6-phosphate deacetylase (PYU1_G006885, EC 3.5.1.25), which contribute to chitin degradation. Interestingly, these genes are adjacent in the *Py*. *ultimum* genome. Orthologs were detected in *Phytopythium*, *Saprolegnia*, and *Aphanomyces*. These enzymes may be used to assimilate carbon from chitin in soil, but may also contribute to the synthesis of their cell walls [26]. Also present in *Pythium* but not *Phytophthora* spp. was a gene predicted to encode γ-amylase (EC 3.2.1.3).

A gene for a putative nitrilase (EC 3.5.5.1), which hydrolyzes non‐peptide carbon-nitrogen bonds, was detected in three of the five sequenced *Pythium* spp. including *Py*. *ultimum* (*e*.*g*. PYU1_G001840), *Phytopythium vexans*, and five of the eleven sequenced *Phytophthora* spp. but not *Ph*. *infestans*. Such enzymes have been shown to break down the plant defense compound hydrogen cyanide and was identified in *Ph*. *ramorum* as a candidate for horizontal gene transfer [27, 28]. Its patchy distribution in *Pythium* and *Phytophthora* suggests that the gene may have been present in an oomycete ancestor.

### Overview of metabolic gene expression during tuber colonization

To compare the metabolic transcriptomes of *Ph*. *infestans* and *Py*. *ultimum*, we used RNA-seq data from our prior study that focused on the early and late stages of potato tuber colonization [19]. As shown in Panel A of S2 Fig, *Py*. *ultimum* causes massive host necrosis throughout the disease cycle while necrosis only occurs at the late stages of colonization by *Ph*. *infestans*. The sampling scheme used for early and late timepoints in the RNA-seq experiment is illustrated in Panels B and C of S2 Fig. For *Ph*. *infestans*, samples collected at 1.5 and 4 days after inoculation represented the early and late stages of infection. We previously showed that mRNA levels of markers such as *Avr3A* and *NPP1* were consistent with those timepoints representing biotrophic (early) and necrotrophic (late) stages of growth. For *Py*. *ultimum*, the early and late timepoints were represented by bands within lesions that had been colonized for 0.5 and 1.5 days; both exhibited massive necrosis. The RNA-seq experiment employed three biological replicates, and RT-qPCR of 12 genes showing differential expression in RNA-seq confirmed the robustness of the data [19]. Expression data were also generated from young and older (sporulating) cultures grown on rye and pea broth.

**Fig 2:**
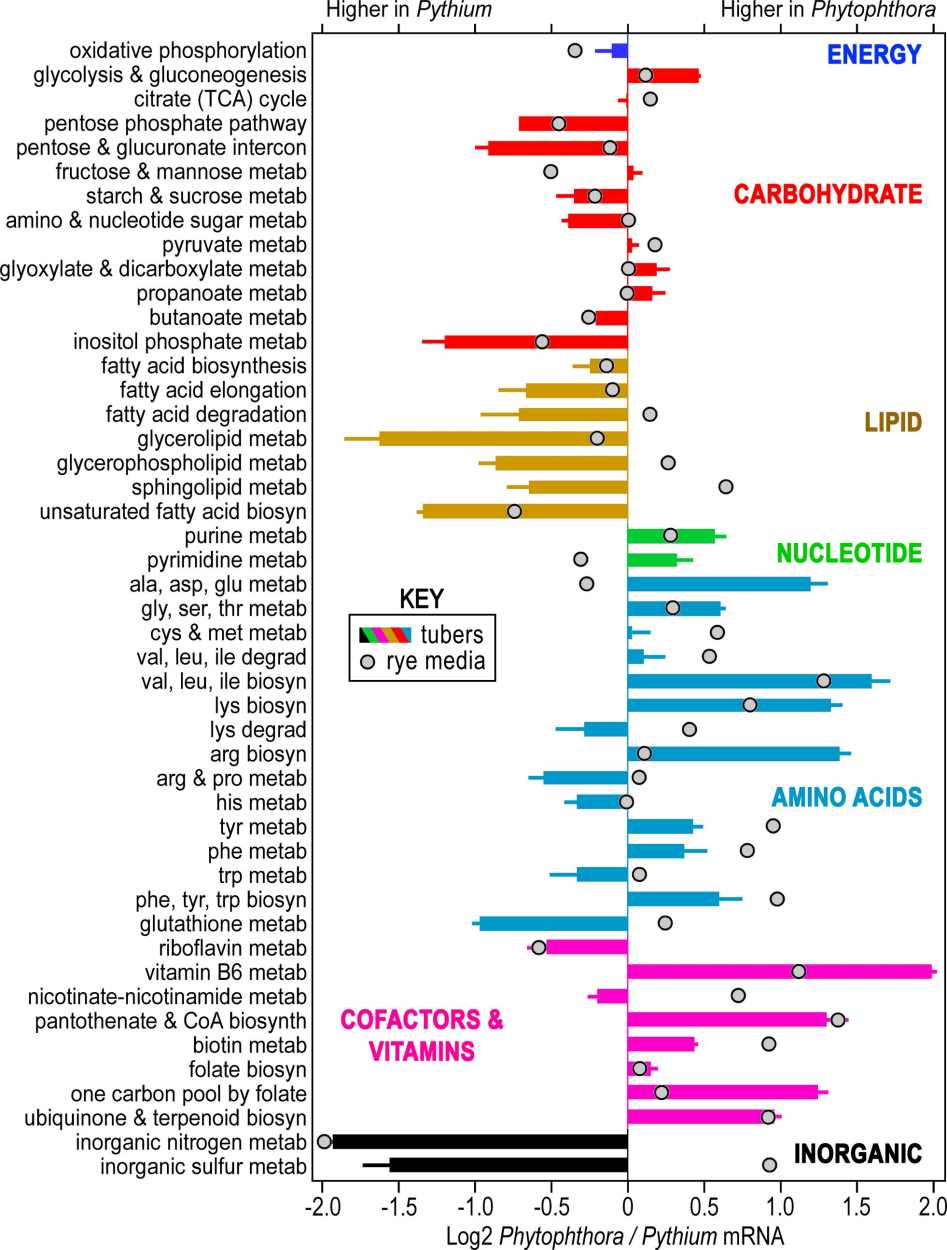
Comparison of expression of metabolic genes during tuber infection and growth on artificial media by *Ph*. *infestans* and *Py*. *ultimum*. Graphed is the log2 ratio of the summed FKPM of genes for each species. Genes were grouped based on pathway maps in the KEGG database, except for genes involved in the assimilation of inorganic nitrogen and sulfur which are listed separately. Bars represent the ratio of expression in the two species at the early infected tuber timepoint, with bars pointing to the right representing higher expression in *Ph*. *infestans*. Each bar is color-coded by category (*e*.*g*. carbohydrate, lipid, nucleotide, amino acid cofactor/vitamin, and inorganic sulfur or nitrogen metabolism). Error bars are based on three biological replicates. Circles represent the expression ratios in the early rye media timepoint.

An overview of metabolism was obtained by summing the FPKM of genes in each of 47 pathways (Fig 2). During early tuber infection, many pathways exhibited higher aggregate expression in one species than the other; "early" in this context corresponds to the biotrophic growth of *Ph*. *infestans* at 1.5 dpi and necrotrophic growth by the faster-growing *Py*. *ultimum* at 0.5 dpi. For example, genes in pathways for inositol phosphate, glycerolipid, and inorganic nitrogen metabolism were transcribed at 2, 3, and 4-fold higher levels in *Py*. *ultimum* (bars in Fig 2). In contrast, most pathways involved in amino acid, nucleotide, and cofactor biosynthesis were expressed 2 to 3-fold higher in *Ph*. *infestans*.

The expression ratios measured between the species *in planta* were generally distinct from the ratios observed in hyphae of similar age from rye media (circles in Fig 2). While most ratios on artificial media were closer to unity than on tubers, this was not always true. For example, many pathways related to forming amino acids were higher in *Ph*. *infestans* in both tubers and media. Similarly, fatty acid biosynthesis was higher in *Py*. *ultimum* under both conditions. A few pathways exhibited opposite biases, such as sphingolipid biosynthesis which was higher in *Py*. *ultimum* on tubers but lower in rye media. It was interesting that the expression of genes related to oxidative phosphorylation varied between the species by less than 30% in both tubers and media.

The biological relevance of the results from the rye media cultures is unclear since this is not a natural growth condition, especially for *Ph*. *infestans*, which only colonizes living plants. Nevertheless, comparisons between gene expression in tubers and media demonstrate that the two species adapt their metabolism to their environment. Similar conclusions can be drawn from RNA-seq data from pea media (S1 Fig).

### Metabolite levels are similar between the species in media

**Fig 3:**
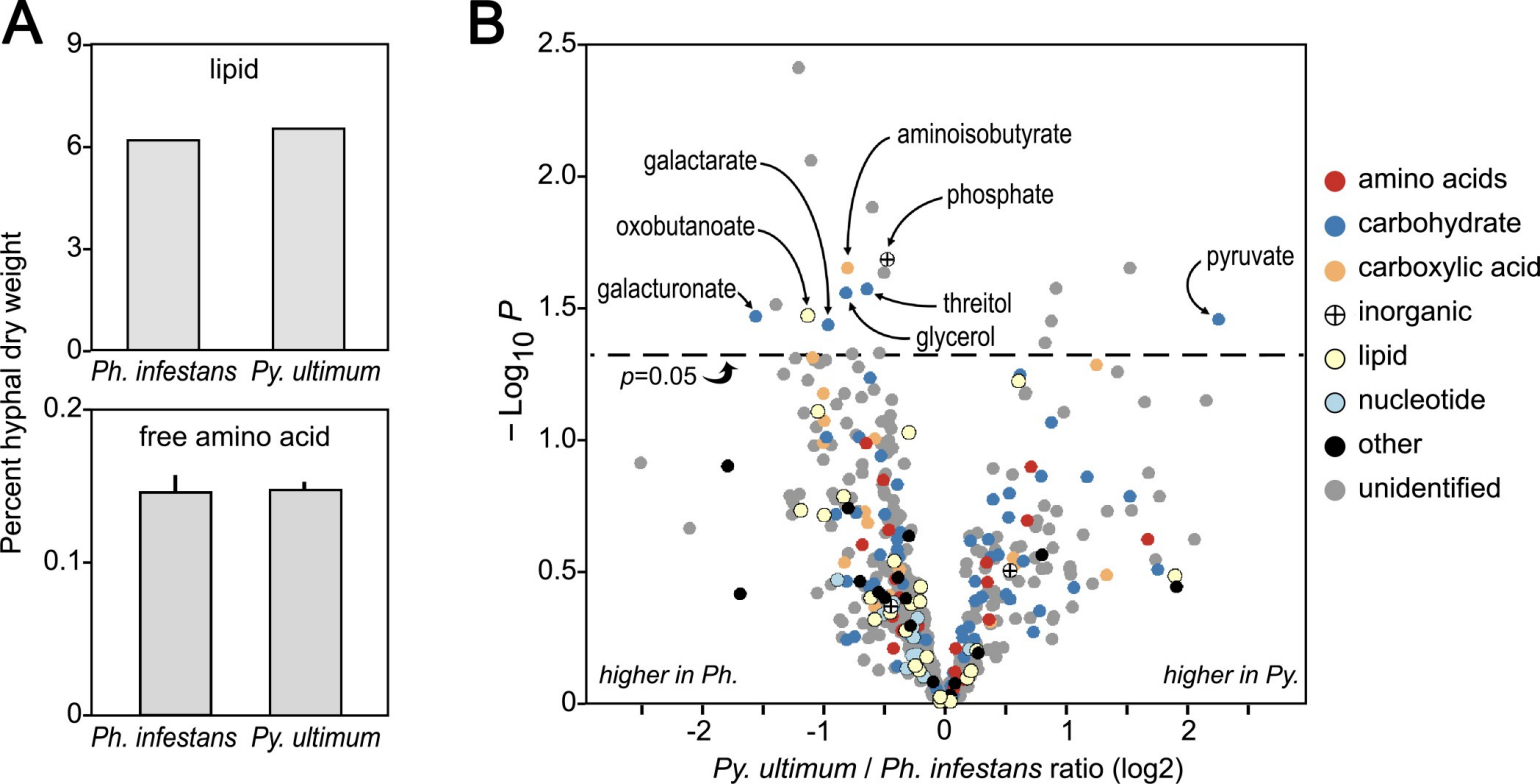
Comparison of metabolite levels in *Ph*. *infestans* and *Py*. *ultimum* hyphae. **(A)** Levels of total lipid and free amino acids in hyphae from rye media. **(B)** Volcano plot comparing metabolite levels. The X-axis indicates the ratio of the abundance in *Py*. *ultimum* compared to *Ph*. *infestans*, based on 12 biological replicates. The Y-axis indicates the *P*-value for differential expression, uncorrected for false discovery due to multiple testing. Marked above the dotted lines are compounds differentially present at *P*<0.05. Compounds are color-coded by category, with unidentified compounds shown in gray.

As described below, several of our experiments suggested that the interspecific variation in mRNA levels associated with pathways was not accompanied by major differences in metabolite pools. For example, although differences were observed in the expression of amino acid and lipid metabolism genes, assays of hyphae from rye media revealed that each species had similar lipid and amino acid contents (Fig 3A).

Moreover, untargeted metabolomics identified only a few differences between young hyphae of *Ph*. *infestans* and *Py*. *ultimum* grown on rye media. This analysis yielded data on 188 identified and 349 unidentified compounds. Based on 12 biological replicates, several differences were detected at an uncorrected *P*-value threshold of 0.05 (Fig 3B). These included four-fold higher pyruvate in *Py*. *ultimum*, and smaller increases in *Ph*. *infestans* of glycerol, threitol, oxobutanoate and aminoisobutyrate (intermediates in amino acid metabolism), and galacturonic acid and galactarate (from pectin degradation). It is tempting to link these differences with our previous data. For example, the higher pyruvate in *Py*. *ultimum* might be attributed to its lack of pyruvate phosphate dikinase (EC 2.7.9.1). Similarly, the higher levels of galacturonic acid and galactarate in *Ph*. *infestans* might reflect the fact that it produces more polygalacturonases (EC 3.2.1.15) than *Py*. *ultimum*, as described in ref. [19]. Such speculations are interesting, but note that no metabolites were significantly different at an FDR threshold of 0.05 based on a Benjamini-Hochberg multiple testing correction. This is consistent with the concept that *Ph*. *infestans* and *Py*. *ultimum* form similar biosubstances through distinctly tuned metabolic pathways.

### Starch metabolism during tuber infection

Potato starch represents about 80% of the carbohydrate of potato tubers, and occurs as intracellular granules comprised of 20% amylose, which are α-glucose units joined by α-1,4 bonds, and 80% amylopectin, which also contains α-1,6 linkages [29, 30]. *Ph*. *infestans* and *Py*. *ultimum* both encode α-amylases (EC 3.2.1.1), which degrade the 1,4 linkage [31]. However, only *Py*. *ultimum* produces an γ-amylase (EC 3.2.1.3), which is needed to break the 1,6 bond. Neither makes a β-amylase (EC 3.2.1.2), which plants and some microbes use to remove disaccharides from starch. Interestingly, our search of other oomycete genomes indicate that only *Saprolegnia* spp. express β-amylase.

**Fig 4:**
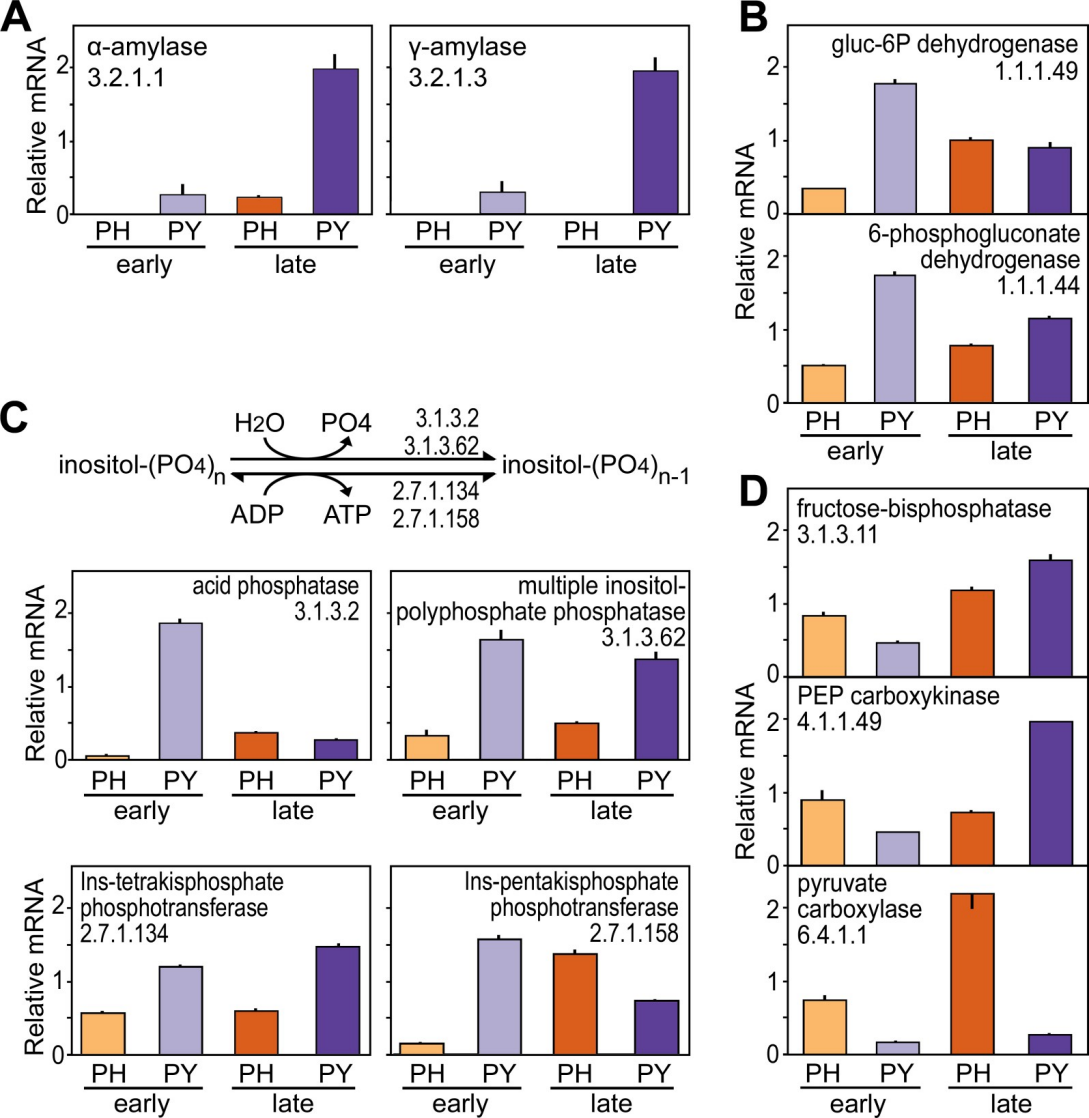
Expression of *Ph*. *infestans* and *Py*. *ultimum* genes involved in starch and inositol phosphate degradation, the pentose phosphate pathway, and gluconeogenesis during tuber infection. **(A)** Genes encoding α-amylase (left) and γ-amylase (right). From left to right, the bars in each graph represent early tuber infection by *Ph*. *infestans* (PH, orange bar), early tuber infection by *Py*. *ultimum* (PY, light purple), late tuber infection by *Ph*. *infestans* (red), and late tuber infection by *Py*. *ultimum* (dark purple). Transcript levels are expressed as relative mRNA values based on the aggregate FPKM of genes in each category, with the mean equaling 1.0. **(B)** Genes encoding rate-limiting steps in the pentose phosphate pathway. **(C)** Genes that remove phosphate from phytic acid and other inositol phosphates. These include two activities that release phosphate (EC 3.1.3.2 and EC 3.1.3.62) and two that convert ADP to ATP (EC 2.7.1.134 and EC 2.7.1.158). **(D)** Genes encoding rate-limiting steps in gluconeogenesis.

The expression patterns of the *Ph*. *infestans* and *Py*. *ultimum* amylases during tuber infection are shown in Fig 4A. In this and subsequent figures, mRNA levels are presented as per-gene normalized values averaged to 1.0, based on the summed FPKM values of genes with the same EC number. At the early stage of infection, α-amylase mRNA was >50 times more abundant in *Py*. *ultimum* than *Ph*. *infestans* (significantly different at *P*<0.05). Expression rose in both species at the late timepoint, particularly in *Ph*. *infestans* where a >50-fold increase was recorded. These patterns are consistent with the predicted abilities of the pathogens to access starch: while *Py*. *ultimum* lyses cells and thus could access starch granules throughout infection, host cell lysis only occurs near the end of the *Ph*. *infestans* disease cycle.

### Gluconeogenesis and pentose phosphate pathway during tuber infection

The high amylase activity of *Py*. *ultimum* during early infection is expected to provide that species with access to more glucose than *Ph*. *infestans*. Consistent with this is our observation that genes encoding glucose-6-phosphate dehydrogenase (EC 1.1.1.49) and 6-phosphogluconate dehydrogenase (EC 1.1.1.44) were expressed at about four-fold higher levels in *Py*. *ultimum* compared to *Ph*. *infestans* during early tuber infection (Fig 4B). These differences were significant at *P*<0.05. The two enzymes represent rate-limiting steps within the oxidative phase of the pentose phosphate pathway, which is an alternative route for obtaining energy from glucose in addition to enabling pentose synthesis. Expression levels of the genes in the two species became similar during late infection.

Conversely, each of the three rate-limiting steps in gluconeogenesis were expressed two to four-fold higher in *Ph*. *infestans* than *Py*. *ultimum* during early tuber infection (Fig 4D). These differences were significant at *P*<0.05. Expression of the genes encoding these enzymes (EC 3.1.3.11, EC 4.1.1.49, and EC 6.4.1.1) at higher levels in *Ph*. *infestans* compared to *Py*. *ultimum* is consistent with its need to generate intermediates for processes such as cell wall and amino acid biosynthesis. *Ph*. *infestans* is also probably better-suited to gluconeogenic flux since it, but not *Py*. *ultimum*, encodes pyruvate phosphate dikinase. This pyrophosphate-dependent transferase converts pyruvate to phosphoenolpyruvate more readily than pyruvate kinase, which uses ATP as the phosphate donor with a much higher ΔG [25].

### Inositol phosphate metabolism during tuber infection

An analysis of genes within this KEGG pathway revealed that the higher level of mRNA scored for *Py*. *ultimum* compared to *Ph*. *infestans* during early infection was attributable to genes that encode enzymes predicted to remove phosphate from inositol hexakisphosphate (InsP6, phytic acid) and partially dephosphorylated derivatives such as inositol tetraphosphate (InsP4) and pentaphosphate (InsP5). Enzymes for metabolizing *myo*-inositol were expressed at similar levels in the two species. About 20% of the total phosphate in tubers is stored as InsP6 [31], primarily in protein storage vacuoles [32]. These organelles are expected to be accessed by *Py*. *ultimum* throughout disease but only during late infection by *Ph*. *infestans*.

As shown in Fig 4C, this predicted pattern of InsP6 accessibility by the pathogens matches the expression of genes encoding InsP4 phosphotransferase (EC 2.7.1.34), InsP5 phosphotransferase (EC 2.7.1.158), multiple inositol-polyphosphate (InsP4 to P6) phosphatase (EC 3.1.3.32), and acid phosphatase (EC 3.1.3.2). For example, in early tubers these genes exhibited 2.5 to 50-fold higher expression in *Py*. *ultimum*, with the differences being significant at *P*<0.05. Increases were also observed between early and late infection by *Ph*. *infestans*, especially for InsP5 phosphotransferase which accumulated nine-fold higher levels of mRNA. Oomycetes lack orthologs of the InsP6 phytases that occur in plants and some microbes (EC 3.1.3.8 and EC 3.1.3.26), but some EC 3.1.3.2 phosphatases are known to act as phytases [33].

### Glycerolipid metabolism during tuber infection

**Fig 5:**
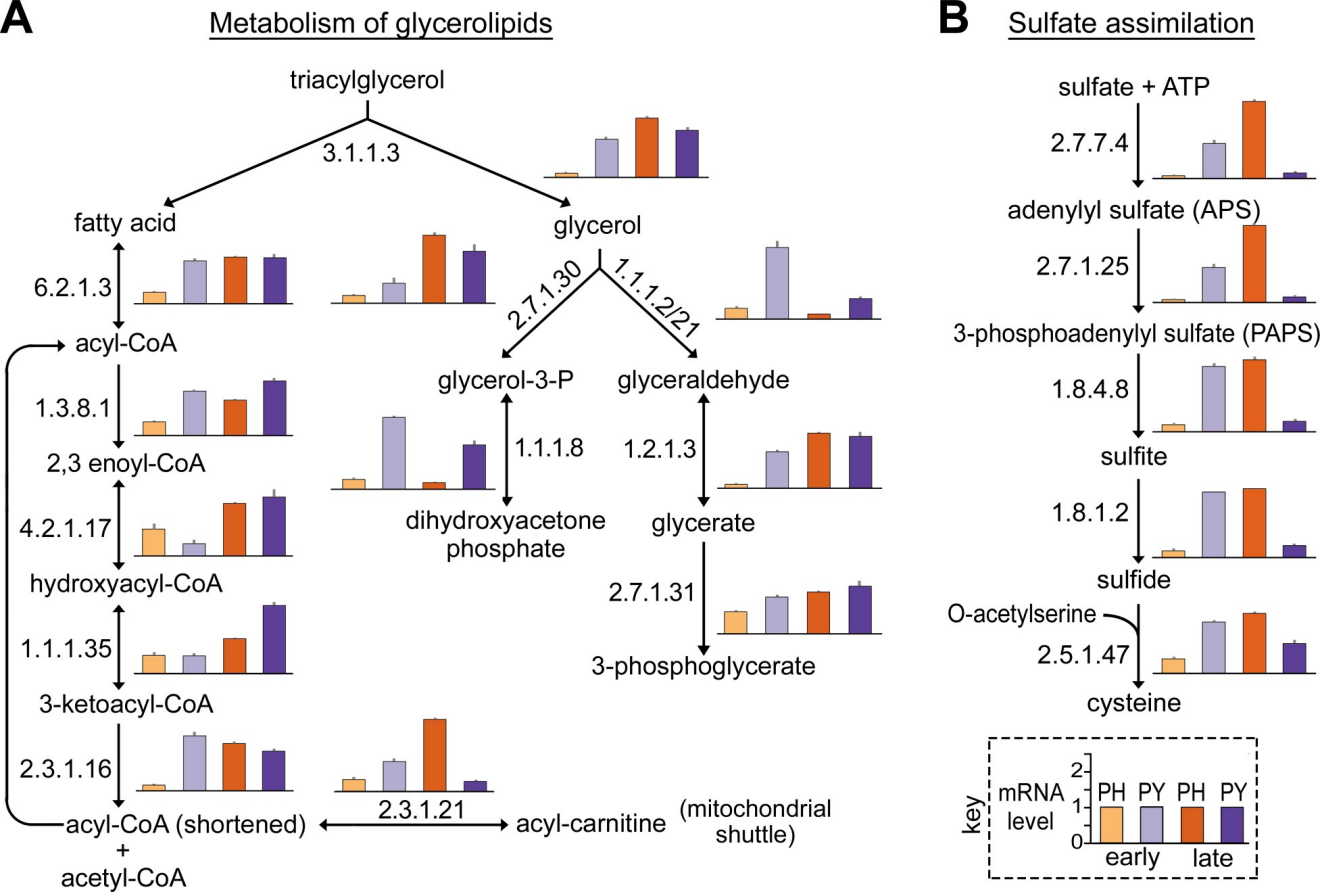
Expression of *Ph*. *infestans* and *Py*. *ultimum* genes involved in glycerolipid metabolism and sulfate assimilation during tuber infection. **(A)** Relative mRNA levels of genes involved in glycerolipid metabolism, starting from the cleavage of a triacylglycerol into glycerol and fatty acids. Enzyme Commission protein 2.3.1.21 is part of the carnitine shuttle that moves cytoplasmic acyl-CoA into the mitochondria. **(B)** Genes involved in the assimilation of inorganic sulfate. In both panels, the samples in the bar graphs are indicated in the key, and continue the color scheme and sample orders used in Fig 3. The y-axis in each bar graph represents relative mRNA levels, per-gene normalized to an average of 1.0 for all four samples, as indicated in the key. Indicated in the pathway is each metabolite and the Enzyme Commission (EC) number for each enzyme.

We showed above (Fig 2) that the expression of genes in the KEGG glycerolipid metabolism pathway during early infection was three-fold higher in *Py*. *ultimum* than *Ph*. *infestans*. This was found to be attributable to the transcription patterns of lipases and enzymes for metabolizing the lipase reaction products, glycerol and fatty acids (Fig 5A). For example, the level of lipase mRNA (EC 3.1.1.3) during early tuber colonization was 3-fold higher in *Py*. *ultimum* than *Ph*. *infestans*. Although not shown in the figure, mRNA levels of genes encoding other classes of lipases were also higher in *Py*. *ultimum* during early infection including acylglycerol lipase (EC 3.1.1.23, 2-fold higher) and lysosomal lipases (EC 3.1.1.5 and EC 3.1.1.13, 11 and 6-fold higher, respectively). Five of the six enzymes involved in the β-oxidation of fatty acids also exhibited higher expression in *Py*. *ultimum* in early tubers. This includes the gene encoding the enzyme for the carnitine shuttle that brings acyl-CoA into mitochondria (EC 2.3.1.21). Each of these differences between the species were significant at *P*<0.05. One exception to this pattern was enoyl-CoA hydratase (EC 4.2.1.17), which showed similar mRNA levels in the two species. This bidirectional enzyme both degrades and synthesizes fatty acids.

Each of the five enzymes that convert glycerol to the glycolytic intermediates dihydroxyacetone phosphate and 3-phosphoglycerate were also expressed at higher levels in *Py*. *ultimum* during early tuber infection, such as glycerate kinase (EC 2.7.1.31) and glycerolphosphate dehydrogenase (EC 1.1.1.8). Each of these differences were significant at *P*<0.05. During late infection, the aggregate expression of three of these five enzymes as well as lipases rose in *Ph*. *infestans* to levels similar to that observed for *Py*. *ultimum*, consistent with the transition of *Ph*. *infestans* to necrotrophy. It should be noted that a few of the enzymes in Fig 5A contribute to multiple pathways. For example, acetaldehyde dehydrogenase (EC 1.2.1.3) converts not only glyceraldehyde to glycerate but also participates in the biosynthesis of carnitine. Similarly, some acid phosphatases (EC 3.1.3.2) discussed in the prior section may also act on glycerol phosphates produced during lipid catabolism.

Transcript levels of each enzyme involved in glycerolipid metabolism increased in *Ph*. *infestans* during late tuber infection to levels matching or exceeding that observed in early infection by *Py*. *ultimum*. This may reflect the transition of *Ph*. *infestans* to necrotrophy. An alternative explanation is that this aspect of metabolism increases during sporulation, which occurs during late infection.

### Sulfate assimilation during tuber infection

As shown in Fig 2, the aggregate level of mRNA related to inorganic sulfate metabolism during early tuber colonization was 3-fold higher in *Py*. *ultimum* than *Ph*. *infestans*. Although we are unaware of data from potato tubers, plant leaves contain small amounts of sulfate in the apoplast, typically 0.01 to 1 mM, with much greater concentrations in cells within vacuoles, typically around 25 mM [34].

Further analysis indicated that the imbalance in mRNA levels was due to the pathway shown in Fig 5B, in which sulfate is reduced and converted to cysteine. Each of the five activities, starting at 3'-phosphoadenosine 5-phosphosulfate synthase (EC 2.7.7.4) and ending with cysteine synthase (EC 2.5.1.47), came from genes that were expressed between 3 and 10-times higher in *Py*. *ultimum* than *Ph*. *infestans* during early tuber infection. Each of these differences were significant at *P*<0.05. Similar to the situation in metazoans, our analysis indicates that the first two steps of this pathway in oomycetes are catalyzed by a bifunctional enzyme, phosphoadenosine-phosphosulfate synthase (PAPS), unlike the situation in bacteria and plants which use two separate polypeptides [35]. Continuing the trend seen with the enzymes described earlier, expression of all of the sulfate assimilation genes rose in *Ph*. *infestans* during late infection to levels resembling those of *Py*. *ultimum* during early infection. This is consistent with the transition of *Ph*. *infestans* to necrotrophy. However, the mRNA levels of most genes encoding sulfate assimilation enzymes declined in *Py*. *ultimum* during late infection.

It should be noted that we cannot claim with 100% confidence that the pathway in Fig 5B operates as shown, due to uncertainty about whether oomycetes make O-acetylserine. This substrate is made in most other organisms by serine O-acetyltransferase (EC 2.3.1.30), but no *Ph*. *infestans* or *Py*. *ultimum* gene was computationally predicted to encode that activity. However, two were annotated as homoserine acetyltransferase (EC 2.3.1.31), and one might be the EC 2.3.1.30 protein since enzymes that act on serine and homoserine are difficult to distinguish. Alternatively, a single enzyme might acetylate both serine and homoserine since some bacterial enzymes exhibit a similar K_cat_ for both substrates [36]. Most oomycetes can also transform sulfate to sulfite using sulfite oxidase (EC 1.8.3.1). This enzyme is not shown in the figure but was expressed at slightly higher levels in *Ph*. *infestans*.

### Amino acid biosynthesis during tuber infection

Multiple pathways related to forming amino acids displayed higher aggregate mRNA levels in *Ph*. *infestans* during early tuber colonization compared to *Py*. *ultimum* (Fig 2). In general, amino acids are generated from glycolytic and citric acid cycle intermediates and through interconversions from other amino acids. Tubers are a relatively rich source of free amino acids (50–100 mM), but most reside within the vacuole [37].

**Fig 6:**
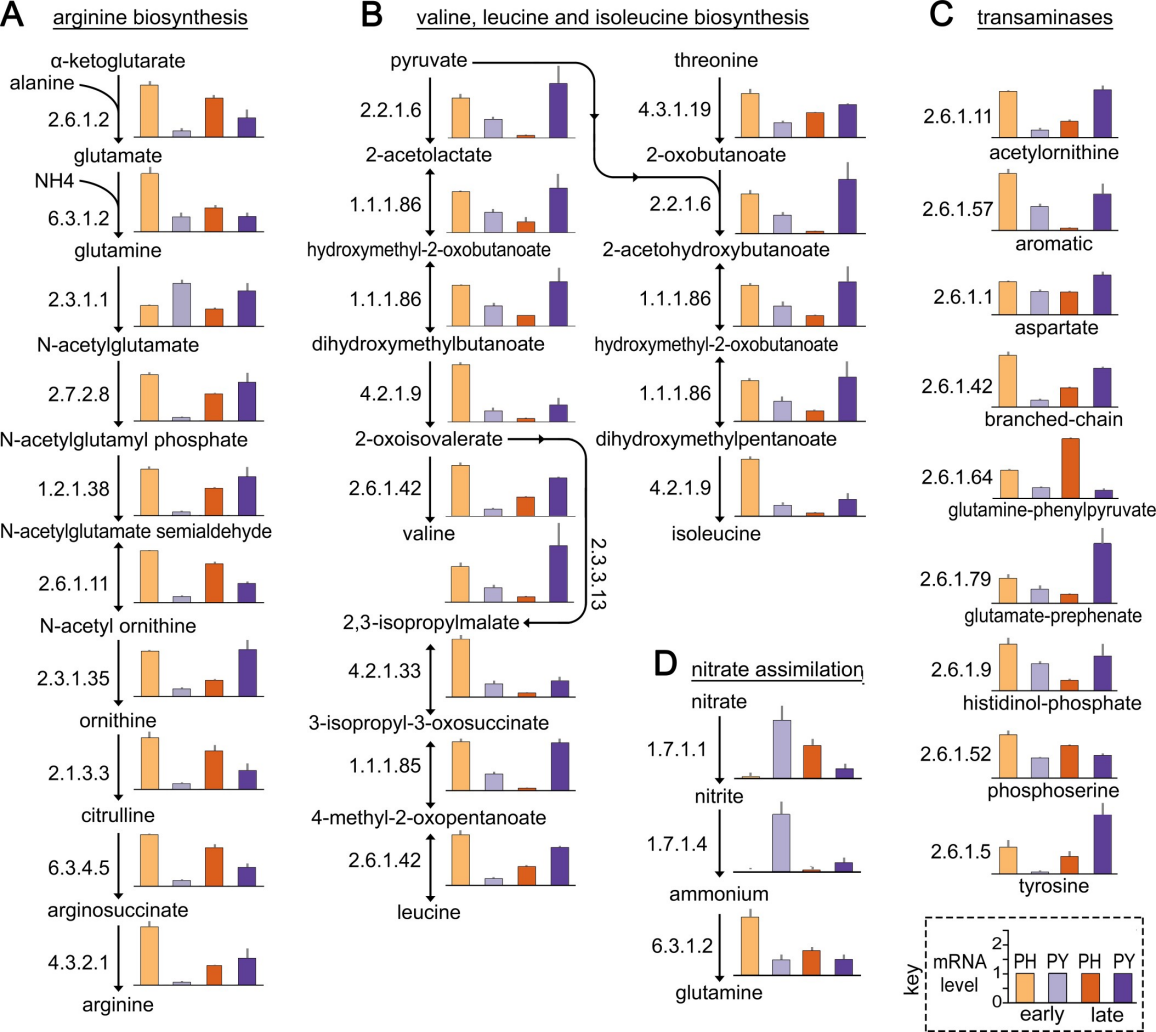
Expression of *Ph*. *infestans* and *Py*. *ultimum* genes involved in the biosynthesis of amino acids during tuber infection. **(A)** Relative mRNA levels of genes participating in arginine biosynthesis. **(B)** Genes involved in the biosynthesis of valine, leucine, and isoleucine. **(C)** Transaminases involved in the biosynthesis of other amino acids. **(D)** Pathway for assimilating nitrate into glutamine. The samples and y-axis scale in the bar graphs are shown in the key, and are the same as in Fig 5.

An examination of the expression data underlying the pathway summaries in Fig 2 indicated that enzymes that make most amino acids, except for methionine, were expressed significantly higher in *Ph*. *infestans* than *Py*. *ultimum* (*P*<0.05) during early tuber colonization. This is illustrated in Fig 6A for arginine and Fig 6B for valine, leucine, and isoleucine. For example, genes encoding nine of the ten enzymes used to convert α-ketoglutarate to arginine were transcribed 5 to 10-fold higher by *Ph*. *infestans*. The only exception was glutamate N-acetyltransferase (EC 2.3.1.1), which had higher expression in *Py*. *ultimum*. In the pathway that generates valine, leucine, and isoleucine, genes for all 14 enzymes exhibited higher mRNA levels in early tuber infection by *Ph*. *infestans* than *Py*. *ultimum*. Interestingly, during late infection mRNA levels for each of these genes rose in *Py*. *ultimum* but fell in *Ph*. *infestans*. This decline in the latter may be related to the general reduction in metabolic mRNAs that we observed previously in *Ph*. *infestans* upon sporulation, since spores were forming at our late timepoint. The rise in *Py*. *ultimum* late during its disease cycle likely indicates that amino acids are becoming depleted.

The transaminases (EC 2.6.1.-) that are used to form all amino acids other than those mentioned earlier are shown in Fig 6C. Genes for these were uniformly expressed at higher levels in *Ph*. *infestans* during early infection (*P*<0.05). In most cases, their mRNAs rose in *Py*. *ultimum* during late infection.

### Nitrate assimilation during tuber infection

Nitrogen for amino acids, nucleotides, and other substances in eukaryotes are usually obtained from another organic compound such as an amino acid. Ammonium and nitrate are other sources, with the latter being assimilated through the action of nitrate reductase (NR; EC 1.7.1.1) and nitrite reductase (NiR; EC 1.7.1.4). The NR and NiR genes, along with one encoding a nitrate transporter (NRT), form a cluster in *Phytophthora* and *Pythium* genomes that appears to have been transferred horizontally from an oomycete ancestor to fungi [38].

Major differences in NR and NiR expression in *Ph*. *infestans* and *Py*. *ultimum* were observed during tuber colonization. As shown in Fig 6D, both enzymes were expressed >50-fold more by *Py*. *ultimum* during early infection. These differences were significant at *P*<0.05. Their transcripts rose during late infection by *Ph*. *infestans*.

These results were intriguing for several reasons. First, most isolates of *Ph*. *infestans* are reportedly unable to grow using nitrate as the sole nitrogen source [39]. This raises the question of whether the assimilation pathway is functional in *Ph*. *infestans*. This is not an unreasonable speculation considering that downy mildews have lost NR and NiR [40]. Second, our assays indicated that tubers contained only about 0.4 mM nitrate, compared to 50–100 mM of free amino acids and 2 mM ammonium. In most other organisms, nitrate assimilation is repressed by amino acids and ammonium since these alternative nitrogen sources do not require energy-intensive reduction steps [41]. It was therefore surprising that *Py*. *ultimum* strongly up-regulated the nitrate assimilation genes in the presence of a 100-fold excess of other nitrogen sources.

### Nitrate does not support *Ph*. *infestans* growth

To confirm prior reports that nitrate is a poor source of nitrogen for *Ph*. *infestans* [42], we tested five isolates of *Ph*. *infestans* along with *Py*. *ultimum* and two isolates of *Phytophthora mirabilis*. The latter is a pathogen of the perennial herbaceous plant *Mirabilis jalapa* (four-o'clock) and is related to *Ph*. *infestans* [39]. Testing all three species on a common defined medium was challenging since most media did not support the growth of all species or strains. A version of Henninger's media [43] with nitrate or ammonium substituting for amino acids proved adequate, although growth was poor and the media did not support sporulation.

**Fig 7:**
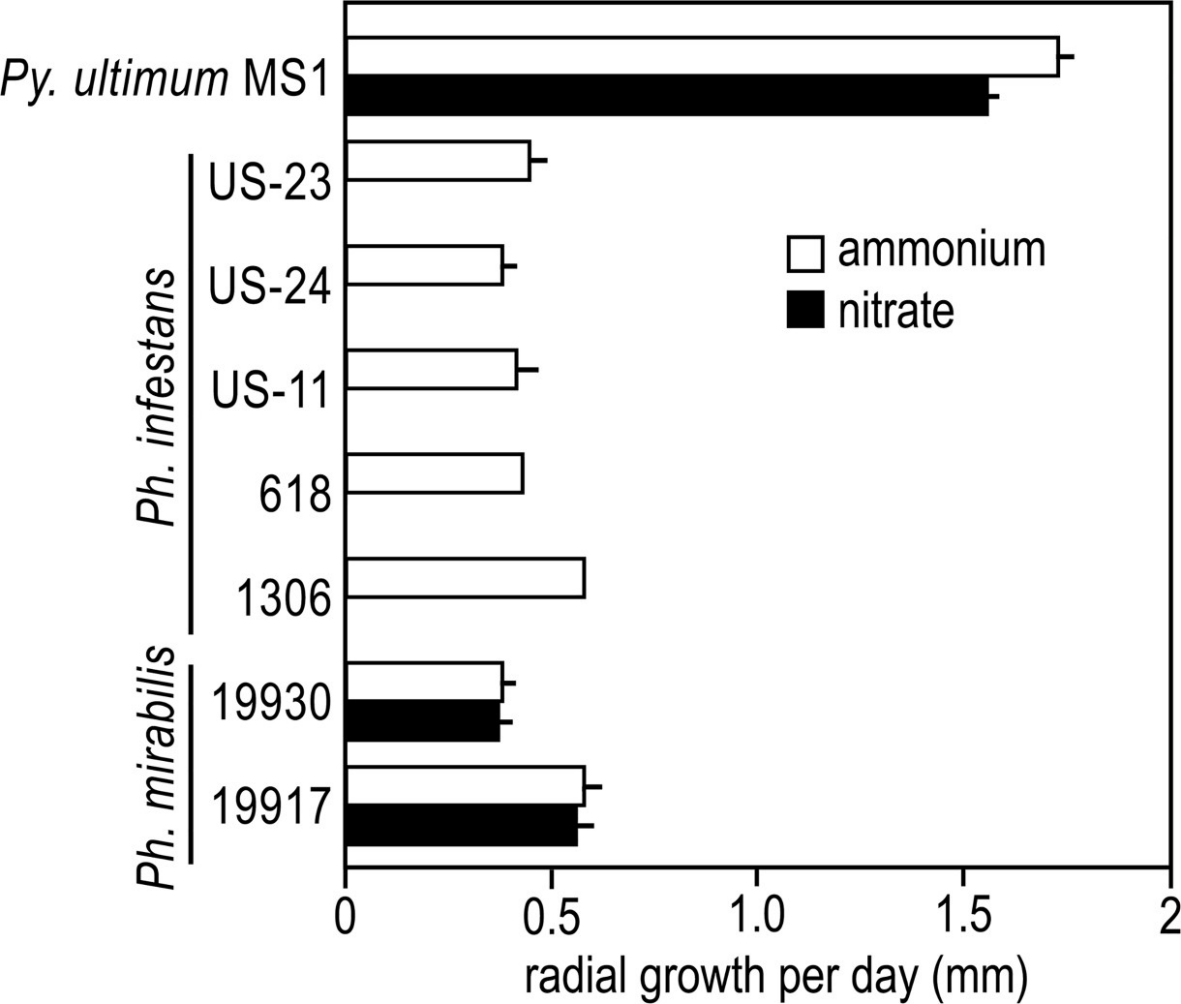
Growth of *Py*. *ultimum*, *Ph*. *infestans*, and *Ph*. *mirabilis* on using ammonium or nitrate as the nitrogen source. Indicated is the rate of radial growth of the indicated isolates on Henninger media modified to use ammonium sulfate or potassium nitrate as the nitrogen source. Error bars represent standard deviation based on three biological replicates.

All species grew in the defined media when the nitrogen source was 10 mM ammonium (Fig 7). *Py*. *ultimum* and *Ph*. *mirabilis* grew at similar rates on ammonium or nitrate. However, none of the *Ph*. *infestans* isolates grew when the ammonium was substituted by nitrate. The same result was obtained with *Ph*. *infestans* regardless of whether potassium or sodium nitrate was used, if the concentration was dropped to 1 mM, or if the pH of the media was reduced.

### The species exhibit major differences in nitrate uptake

**Fig 8:**
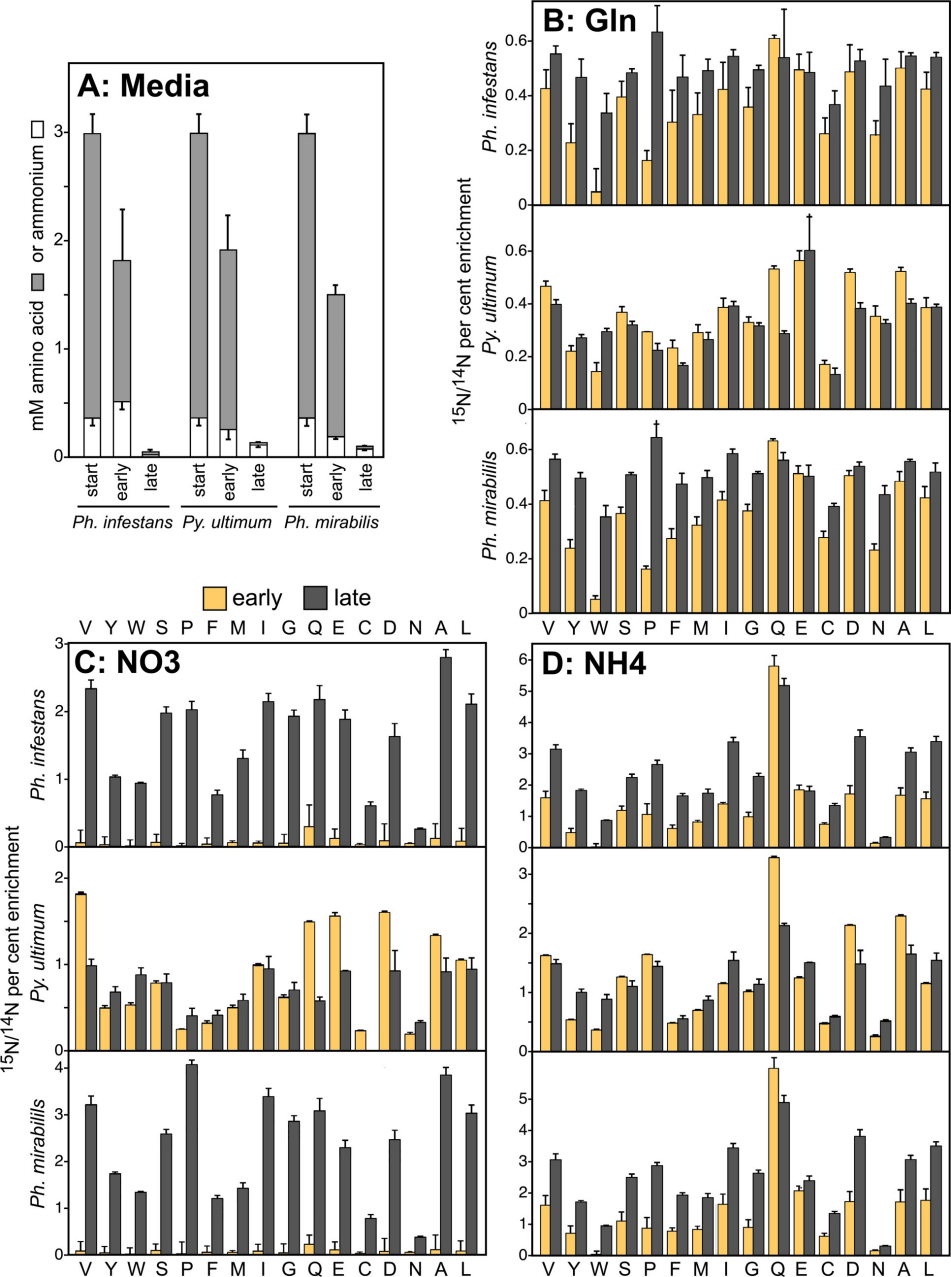
Use of glutamine, nitrate, and ammonium by *Ph*. *infestans*, *Py*. *ultimum*, and *Ph*. *mirabilis*. **(A)** Levels of free amino acids (grey bars) or ammonium (white) in uninoculated clarified rye broth, and in rye broth from early and late timepoints after removing hyphae by filtration. Error bars represent standard deviation based on two replicates. **(B)** Isotopic enrichment using ^15^N-glutamine. Hyphae were collected at the early (light bars) and late timepoints (dark bars) from media supplemented with the ^15^N compound, and the ^15^N/^14^N ratio of amino acids were calculated based on mass spectrometry. Shown under each pair of bars is the corresponding amino acid using a single-letter code. Error bars represent standard deviation based on six biological replicates. **(C)** Same as panel B but using ^15^N-nitrate. **(D)** Same as panel C but using ^15^N-ammonium.

To study nitrate metabolism in the three species, including whether *Ph*. *infestans* assimilated nitrate at all, a labeling study was performed in media spiked with 10 mM ^15^NO_3_^–^, ^15^NH_4_^+^, or ^15^N-Gln. Measurements were made in "early" cultures where amino acids would still be abundant in media, and "late" cultures where amino acids would be depleted. In preliminary experiments, by assaying metabolites remaining in spent broth we established that the "early" and "late" conditions occurred when a hyphal mat initiated from an inoculum plug covered about 20 and 80% of the culture's surface area, respectively. This usually occurred in 2 and 6-day cultures of *Ph*. *infestans*, 3 and 8-day cultures of *Ph*. *mirabilis*, and 1 and 2-day cultures of *Py*. *ultimum*, respectively, although the exact time varied depending on the vigor of the inoculum. As shown in Fig 8A, the early and late cultures of each species retained about 50 and 1% of the starting concentration of amino acids, or about 1.5 and 0.03 mM, respectively. In all experiments described subsequently, spent media was assayed to validate the "early" and "late" condition of each sample. If a culture thought to represent an early timepoint appeared to be too aged based on the residual level of amino acids, it was discarded.

After conditions for "early" and "late" growth were established, the labeling experiment was performed with the three ^15^N-labeled compounds (added to separate cultures) using six biological replicates each, plus unlabeled controls. Based on ^15^N/^14^N ratios in amino acids as measured by mass spectrometry, *Ph*. *infestans* and *Ph*. *mirabilis* both incorporated little ^15^NO_3_^–^ or ^15^NH_4_^+^ in the amino acid-rich early timepoint but used large amounts of these two compounds at the late timepoint (Fig 8C and 8D). For example, about 25 times more ^15^N from nitrate was incorporated into valine at the late versus early stages. ^15^N-Gln was used at similar rates at both timepoints (Fig 8B), while intermediate results were obtained with ^15^NH_4_^+^ (Fig 8D). For example, ammonium was assimilated into valine two times more in the late than early cultures by both *Ph*. *infestans* and *Ph*. *mirabilis*; similar trends were obtained for 13 of the other 14 amino acids that were assayable. The exception was glutamine which acquired nitrogen from ^15^NH_4_^+^ at similar rates at the early and late timepoints; this was not surprising since glutamine synthase was expressed more during early than late infection (Fig 6D; EC 6.3.1.2). The data demonstrate that *Ph*. *infestans* preferentially uses amino acids as a nitrogen source followed by ammonium, has a functional nitrate assimilation pathway, and that the pathway is repressed by amino acids and possibly ammonium similar to what occurs in most fungi [44].

The results with *Py*. *ultimum* were strikingly different. This species incorporated all three nitrogenous compounds at similar rates at each timepoint. Therefore, there was little evidence that amino acids repressed its nitrate assimilation pathway. Since rye media also contains about 0.3 mM ammonium, that compound does not appear to strongly repress nitrate assimilation.

### Nitrate does not induce its assimilation pathway in *Ph*. *infestans*

**Fig 9:**
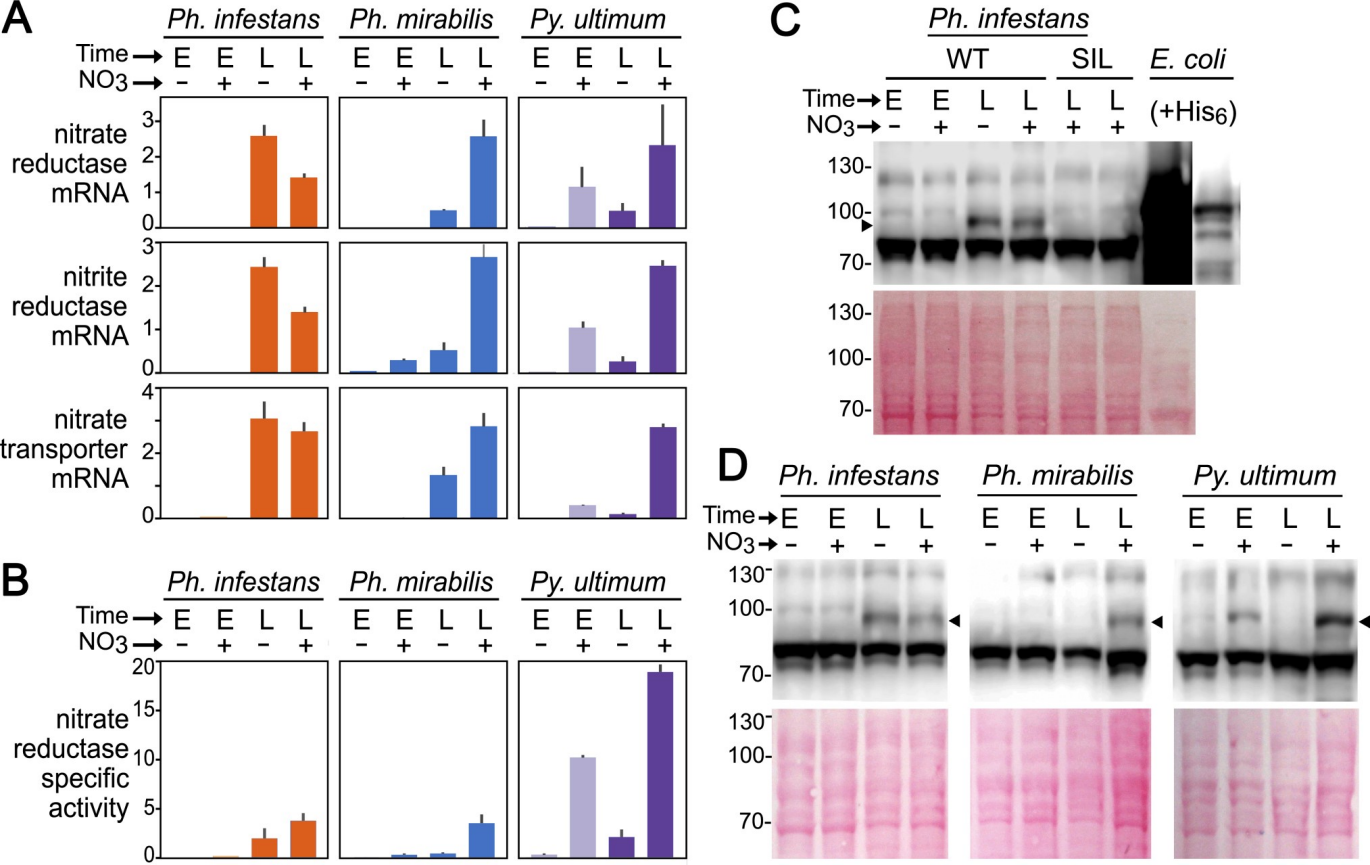
Expression of nitrate assimilation pathway in *Ph*. *infestans*, *Ph*. *mirabilis*, and *Py*. *ultimum*. **(A)** Expression of nitrate reductase, nitrite reductase, and nitrate transporter. The top three rows indicate mRNA levels of the three genes determined by RT-qPCR; data are presented as per-gene normalized values. The tissue samples were taken from early (E) or late (L) timepoints in rye broth media supplemented with 10 mM potassium nitrate (+) or unsupplemented media (-). Error bars represent standard error based on two biological replicates. **(B)** specific activity of nitrate reductase enzyme, expressed as μmoles min^-1^ mg^-1^. (**C)** Nitrate reductase protein in *Ph*. *infestans*. Indicated is a western blot using hyphae from wild-type (WT) or transformants silenced for the nitrate reductase gene (SIL), using tissues from early or late timepoints in rye broth with or without 10 mM potassium nitrate. The two right lanes contain protein from *E*. *coli* expressing the *Ph*. *infestan*s nitrate reductase containing a hexahistidine tag; the right-most lane is a shorter exposure. **(D)** Nitrate reductase protein in *Ph*. *infestans*, *Ph*. *mirabilis*, and *Py*. *ultimum*. The top panel shows a western blot using hyphae from early and late timepoints with and without 10 mM potassium nitrate; the lower panel is a Ponceau-stained membrane. The arrow indicates the position of the nitrate reductase protein.

Another conspicuous difference between the species was that nitrate had a strong stimulatory effect on the expression of nitrate reductase, nitrite reductase, and the nitrate transporter in *Py*. *ultimum* (NR, NiR, and NRT, respectively), little or no effect in *Ph*. *infestans*, and an intermediate effect in *Ph*. *mirabilis*. This is indicated by the RT-qPCR data in Fig 9A. For example, adding 10 mM nitrate to rye media caused NR mRNA in *Py*. *ultimum* to increase by 20 and 5-fold at the early and late timepoints, respectively. Parallel changes were observed for NiR and NRT mRNA. Our assays indicate that unamended rye media contains about 40 μM nitrate.

In contrast, the *Ph*. *infestans* genes were not induced by 10 mM nitrate in media at the early or late timepoints. They were expressed highly at the late timepoint regardless of whether 10 mM nitrate was added. This mirrors their behavior during early and late tuber colonization (Fig 6D). Culture age thus appears to be a strong determinant of expression of the genes in *Ph*. *infestans*.

With *Ph*. *mirabilis*, adding 10 mM nitrate to early media cultures caused about two-fold increases in NR and NRT mRNA, although the absolute level of transcripts was very small (too low to be visible in Fig 9A). At the late timepoint, mRNA levels increased strongly regardless of nitrate concentration, similar to *Ph*. *infestans*. However, adding nitrate caused an additional 2 to 5-fold increase in mRNA levels in the late cultures of *Ph*. *mirabilis*. Interestingly, higher induction was observed for NiR compared to NR, which is similar to the response observed in *N*. *crassa* [45].

### NR expression is regulated mostly at the transcriptional level

Since NR in some organisms is subject to post-translational regulation [46], we compared levels of NR mRNA with its enzymatic activity (Fig 9B). Post-translational events did not appear to be a major factor in regulating the enzyme since NR specific activity and mRNA generally increased in parallel in each of the three species. Nitrate did seem to cause a disproportional increase in enzyme activity in late cultures of *Ph*. *infestans*, however. In the presence of nitrate, the specific activity of NR was about four times higher in *Py*. *ultimum* than *Ph*. *infestans* or *Ph*. *mirabilis*.

The use of an antibody against NR also showed that mRNA and protein levels changed in concert. In our initial tests against protein extracts from *Ph*. *infestans*, we observed that the antibody was not very specific (Fig 9C). NR from *Ph*. *infestans* and *Py*. *ultimum* are both predicted to be 93 kDa, after removing an intron from the *Ph*. *infestans* gene model which appeared to be incorrect based on RNA-seq data. While the antibody detected a 93 kDa band, it also detected a stronger band at 82 kDa. As shown in Fig 9B, we determined that the 93 kDa band was NR since it was absent in two strains in which NR transcription was blocked by homology-dependent gene silencing [47]. Also, the possibility that NR had aberrant electrophoretic mobility seemed unlikely since recombinant *Ph*. *infestans* NR expressed in *E*. *coli* with a 3 kDa hexahistidine tag yielded a band with an apparent mobility of 96 kDa (Fig 9C, right lane).

Next, we extended our analysis to all three species. The pattern of production of NR protein in *Ph*. *infestans*, *Ph*. *mirabilis*, and *Py*. *ultimum* is shown in Fig 9D. The intensity of the 93 kDa band shows a good match to the levels of NR mRNA and enzymatic activity shown in Fig 9A, and confirms that expression of the enzyme is regulated divergently in the three species. For example, NR protein was nitrate-induced in *Py*. *ultimum* and *Ph*. *mirabilis*, but in *Ph*. *infestans* was not induced by nitrate. Nitrate seemed to reduce NR protein (and mRNA; Fig 9A) levels slightly in *Ph*. *infestans*, which could be a sign of nitrate toxicity. In both *Ph*. *infestans* and *Ph*. *mirabilis*, the NR band was stronger in the late compared to early nitrate-supplemented samples. Since the right-most lane in the *Ph*. *mirabilis* panel was overloaded by 2–3 times compared to the other *Ph*. *mirabilis* lanes, we repeated the blot using fresh samples. This confirmed that its NR protein was more abundant in the late cultures, provided that nitrate was present (S3 Fig).

### A genus-specific transcription factor may regulate nitrate assimilation

During our analysis of oomycete genomes we observed that all species of *Phytophthora* as well as downy mildews encode a protein annotated as a fungus-like nitrogen metabolite repression regulator. Intriguingly, this appears to be missing from other oomycetes including all sequenced species of *Pythium*. The fungal protein, which is called NMRA in *Aspergillus nidulans* and NMR1 in *Neurospora crassa*, is a transcription factor that causes more readily assimilated nitrogen sources such as ammonium and glutamine to be used instead of nitrate [44].

**Fig 10:**
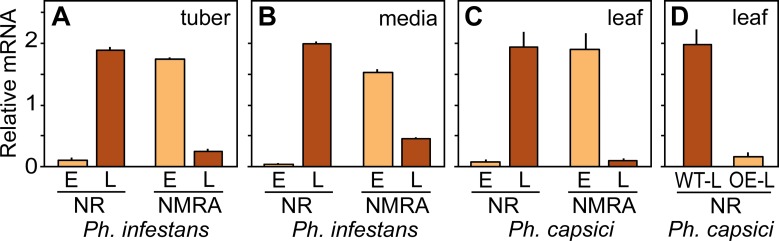
Expression of nitrate reductase and the NMRA transcription factor in *Ph*. *infestans* and *Ph*. *capsici*. **(A)** Relative expression levels in *Ph*. *infestans* of nitrate reductase (NR) and the NMRA-like transcription factor during early (E) and late (L) stages of tuber infection. Data are based on RNA-seq and are normalized to mean equals one. **(B)** Expression in *Ph*. *infestans* of NR and NMRA in rye broth cultures, based on RNA-seq. **(C)** Expression in *Ph*. *capsici* of NR and NMRA at early (E, 8 hr dpi) and late stages (L, 24 hr dpi) of tomato infection, based on microarray analysis. **(D)** Effect of NMRA overexpression on NR transcript levels, based on microarray analysis. Data are from late tomato infection by wild-type *Ph*. *capsici* (WT-L) or a transformant over-expressing NMRA at about 40 times the wild-type level (OE-L).

We observed that NR and the *Ph*. *infestans* ortholog of NMRA, PITG_14492, are expressed in opposing patterns which is consistent with a role of the latter in repressing nitrate assimilation. As shown in Fig 10A, during early tuber colonization NR mRNA was low while NMRA mRNA was high, while the reverse patterns of expression were seen during late tuber infection. The same expression profiles were observed in early and late rye media cultures (Fig 10B). As this paper was being written, a microarray study of *Phytophthora capsici* infecting tomato was published [48]. Our analysis of data from that paper deposited in ArrayExpress showed that the patterns of NR and NMRA expression in *Ph*. *capsici* matched that of *Ph*. *infestans* (Fig 10C). Moreover, an NMRA-overexpressing transformant of *Ph*. *capsici* from that study showed reduced levels of NR mRNA (Fig 10D).

### NR protein has evolved distinct features in each oomycete

**Fig 11:**
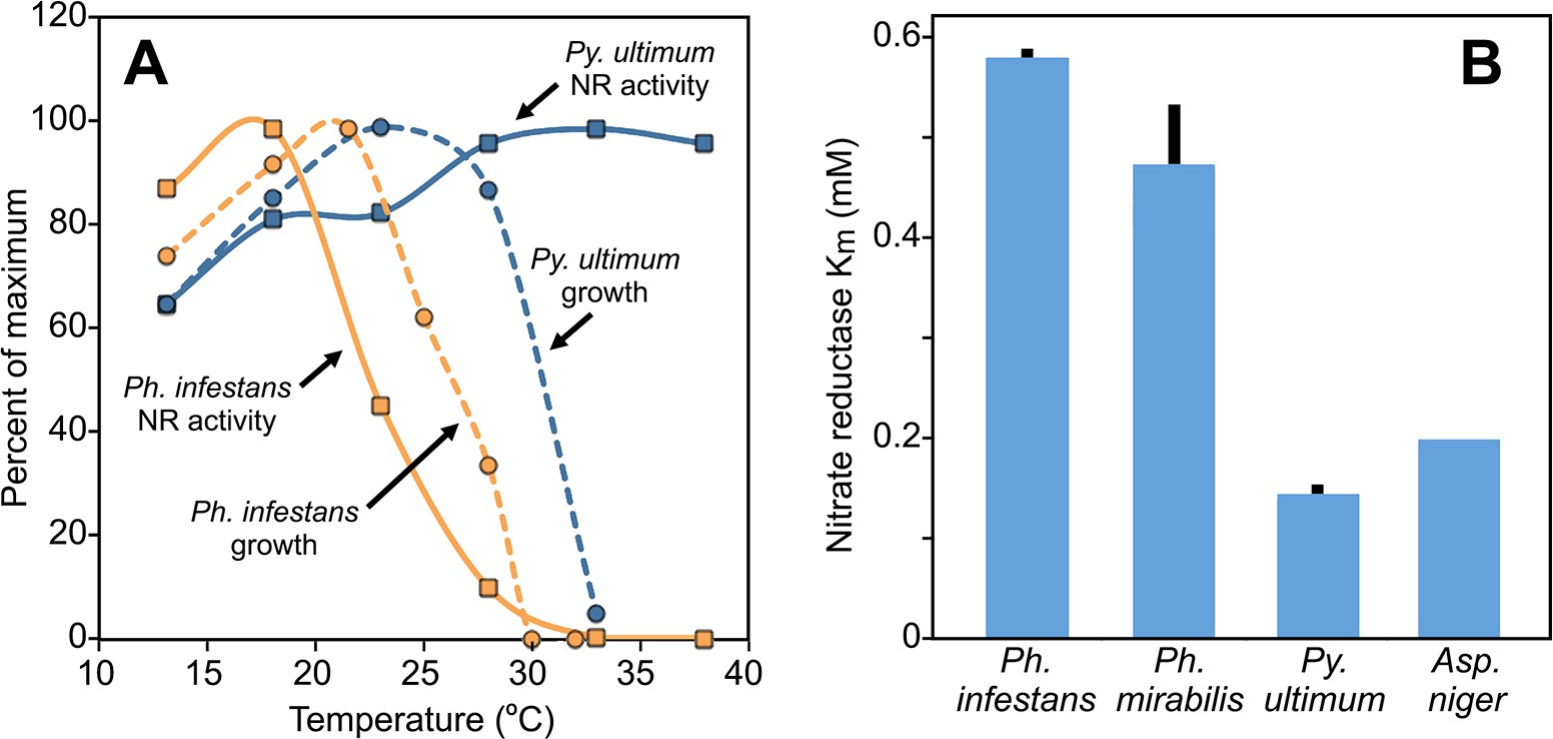
Characteristics of nitrate reductase enzyme. **(A)** Effect of temperature on enzymatic activity of nitrate reductase (NR; solid lines) and growth (dashed lines) of *Ph*. *infestans* and *Py*. *ultimum*. Results are expressed as percent of maximum. **(B)** K_m_ of nitrate reductase from *Ph*. *infestans*, *Ph*. *mirabilis*, *Py*. *ultimum* and *Aspergillus niger*. Bars represent standard deviation from three biological replicates.

We considered the possibility that the NR proteins of each species had acquired distinct characteristics, since the environments that favor their growth are not equal. For example, only *Py*. *ultimum* is able to grow on organic debris in soil. In addition, *Py*. *ultimum* grows over a broader temperature range than *Ph*. *infestans* (Fig 11A, dashed lines). It was therefore interesting to observe that the *Py*. *ultimum* NR remains active at much higher temperatures than NR of *Ph*. *infestans* (Fig 11A, solid lines).

Measurements of the Michaelis constant indicated differences in the affinity of each enzyme for nitrate (Fig 11B). We measured the K_m_ of NR from *Aspergillus niger* at 202 mM, which is close to the published value [49]. Compared to this benchmark, the K_m_ of NR from *Py*. *ultimum* was slightly lower at 146 μM, while both *Ph*. *infestans* and *Ph*. *mirabilis* were much higher at 587 and 469 mM, respectively.

To place these characteristics in context with the sequence similarity of the proteins, we assembled the *Ph*. *mirabilis* gene for NR from short reads deposited at NCBI (Bioproject PRJNA52429). The *Ph*. *infestans* and *Ph*. *mirabilis* proteins exhibited 96% similarity. Both were 76% similar to *Py*. *ultimum* NR, and 55–56% similar to the NR of *A*. *niger*. The *Py*. *ultimum* and *A*. *niger* enzymes exhibited 55% similarity.

## Discussion

We observed variation between the metabolism of *Ph*. *infestans* and *Py*. *ultimum* at multiple levels. Transcriptional differences often reflected each pathogen's access to nutrients. During early tuber colonization, most pathways that were expressed more in *Py*. *ultimum* involved metabolites liberated from lysing plant cells, while those transcribed at higher levels in *Ph*. *infestans* were associated with synthesizing compounds that probably occur at insufficient levels in the apoplast to support growth. A second level of divergence was in the machinery that regulates metabolism, such as that pertaining to nitrate assimilation which in *Py*. *ultimum* was more substrate-responsive and less repressed by other nitrogen sources. Regulatory differences may also affect amino acid biosynthesis, since most of these pathways were expressed at higher levels in *Ph*. *infestans* than *Py*. *ultimum* both during growth in media and tubers. A third facet of variation involved species-specific genes, such as γ-amylases which occur only in *Py*. *ultimum* and presumably help it exploit the most abundant carbon source in tubers. A fourth level of variation concerned protein function, as evidenced by the divergent K_m_ and temperature optima of the NRs. Thus, some interspecific differences were due to short-term physiological responses and others to long-term evolutionary changes.

We also observed that many pathways in *Ph*. *infestans* shifted to a higher *Pythium*-like pattern of expression during late infection. For example, transcripts of most glycerolipid, sulfate, and starch metabolism genes in *Ph*. *infestans* rose at the late timepoint. We believe that this is the first firm evidence that *Ph*. *infestans* perceives and utilizes nutrients from lysing cells. Although the textbook description of *Ph*. *infestans* is as a hemibiotroph, necrotrophy is not equivalent to necrogeny. Whether behavior that leads to plant death is necessarily necrotrophic has been debated in several pathosystems [50]. Alternative hypotheses have included the possibility that host necrosis is not a trophic strategy but instead reflects an inability of older hyphae to deliver defense-blocking effectors, or serves to humidify the apoplast [51].

Many of our conclusions are based on studies of mRNA levels, which might not always match enzymatic activity due to post-transcriptional or translational control [52]. Metabolic flux is also a function of substrate and not just enzyme concentration [53], and many enzymes belong to complex networks. For example, while the higher expression of glycerolipid-degrading enzymes by *Py*. *ultimum* during early tuber infection is consistent with its need to digest host membranes to access intracellular nutrient, *Py*. *ultimum* may also be more inclined to cycle its own carbon through lipids.

To test whether measurements of mRNA levels were a good surrogate for pathway activity, we focused on nitrate assimilation. We found parallel changes in NR mRNA, protein, enzymatic activity, and nitrate incorporation in both species. This concordance might not hold for all pathways, however. Indeed, differences in the levels of mRNAs for metabolite biosynthesis between the species were usually not reflected in the metabolite concentrations that were measured in media. While some of this disparity might be due to post-transcriptional control, it seems that *Ph*. *infestans* and *Py*. *ultimum* use distinct blends of metabolic pathways to form the same building blocks for growth.

While some mRNA patterns were caused apparently by substrate-level induction of transcription, we observed higher expression of amino acid biosynthesis genes in *Ph*. *infestans* in both tubers and rye media. This may be an evolutionary adaptation that reflects the intimate interaction between *Ph*. *infestans* and its hosts. In plants, most amino acids enter the apoplast through UMAMITs (Usually Multiple Amino acids Move In and out Transporters; [54]). However, some amino acids have very low apoplastic concentrations and would thus need to be synthesized by *Ph*. *infestans* [55, 56]. While apoplastic amino acids are known to increase during some fungal and bacterial infections, whether this happens with *Ph*. *infestans* is unknown [55, 56]. It would be interesting to investigate whether *Ph*. *infestans* effectors alter source-sink relationships or increase apoplastic nutrients by acting on UMAMITs, similar to how bacterial effectors influence SWEET transporters [57]. Such effects may extend to other compounds such as organic or inorganic phosphates. While intracellular host compounds such as phytate may provide *Py*. *ultimum* with ample phosphorus, studies of *Fusarium graminearum* colonizing maize showed that phosphate levels in the apoplast are growth-limiting [58], a situation that may also affect *Ph*. *infestans*.

Some features of *Py*. *ultimum* metabolism may help it inhabit multiple niches. This species differs from *Ph*. *infestans* in being able to grow not only on plants but also in organic debris in soil, where nitrate is usually present [59]. The low K_m_ of *Py*. *ultimum* NR may help it acquire nitrate even at low concentrations. This resembles the situation in diatoms where K_m_ differences between *Skeletonema* and *Thalassiosira* NRs were linked to their abilities to grow in low-nitrogen environments [60]. A low K_m_ may not benefit *Ph*. *infestans* since that species only colonizes healthy plant tissues, which provide a reliable supply of nutrients. Indeed, we have attempted to infect tomato plants cultured under low-nitrogen regimes with *Ph*. *infestans* but observed little pathogen growth. This resembles the case in *Arabidopsis* where host mutations that reduced amino acid levels blocked downy mildew infection [61].

Another ecological difference between *Py*. *ultimum* and *Ph*. *infestans* is that the former is a pioneer colonizer [62]. Such species outcompete other microbes by growing quickly. *Py*. *ultimum* grows rapidly, but not necessarily efficiently. This may explain why it expressed NR and NiR at similar rates in early cultures (containing substantial amino acids and ammonium) and older nutrient-depleted cultures, and why it incorporated nitrate equally in standard rye media and media supplemented with ammonium or glutamine. In contrast, most fungi and *Ph*. *infestans* preferentially use amino acids or ammonium to avoid expending energy to reduce nitrate [41].

Comparisons of nitrate utilization in oomycetes and fungi are thought-provoking due to the evolutionary history of the pathway, and since NR has been a model for understanding fungal gene regulation. Fungi and oomycetes share little taxonomic affinity as they belong to different kingdoms, but fungi are thought to have acquired the nitrate assimilation gene cluster from an oomycete ancestor [38]. The transfer apparently also involved the nitrogen metabolite repression regulator transcription factor NMRA, which has apparently been deleted from the *Pythium* lineage. The loss of repressors may aid pioneer colonizers by helping them to rapidly use all nutrients in their environment. Also, NMRA may provide little benefit to *Pythium* since amino acids are typically present at low levels in soil. Apparently for similar reasons, soil-inhabiting streptomycetes have rarely developed amino acid feedback repression systems for many biosynthetic pathways [63].

Divergence in the Michaelis-Menten kinetics of NR was an additional interesting finding of the study, and is consistent with emerging evidence that changes in the K_m_ of metabolic enzymes can influence the growth characteristics of an organism [64]. The K_m_ of *Py*. *ultimum* NR resembles that of many fungi [49], which is logical since both are common soil inhabitants. In contrast, the K_m_ from *Ph*. *infestans* and *Ph*. *mirabilis* was several times higher. It follows that *Py*. *ultimum* is better-suited for utilizing low levels of nitrate in the environment. This is congruent with the greater reliance of *Phytophthora* spp. on organic nitrogen, but an alternative theory is that their NRs evolved to help detoxify high concentrations of nitrate, which can reach ~500 mM in leaf sap [65]. A role of NR in detoxification was suggested by our earlier study in *Ph*. *infestans*, in which knockdowns of the NR gene impeded growth and pathogenicity, but only when nitrate was at high levels [47]. *Pythium* mutants unable to grow on nitrate have not been reported.

Why *Ph*. *infestans* lost the ability to grow on nitrate is enigmatic. *Phytophthora fragariae* and *Phytophthora megasperma* also require organic nitrogen, unlike most members of the genus as well as *Pythium* spp. [42]. Perhaps too much use of the assimilation pathway in some species would cause an imbalance in the cellular levels of its pyrimidine nucleotide cofactors. The ability of these species to use nitrate may also have evolved in response to how that compound is mobilized in their hosts. Alternatively, balancing metabolism away from nitrate may have been an energy-saving measure to help the hemibiotrophs compete against plant defenses. This may also explain why biotrophic oomycetes and fungi have lost the nitrate assimilation pathway [40].

One technical lesson from our experience is the importance in metabolic studies of evaluating the status of a culture by assaying nutrients in the media. Inferences about the biology of plant or animal pathogens as well as other microbes are often drawn from comparing gene expression in different media, such as complex or minimal formulations [66, 67]. However, false conclusions may be drawn if nutrient concentrations at harvest time are not known. A related topic that could enhance our understanding of metabolic regulation concerns nutrient reserves in oomycete hyphae. We observed that biomass continued to increase in older cultures even after nutrients such as free amino acids were exhausted from the media. This suggests that growth is being sustained at least partly through the use of reserves. While the roles of lipid, protein, and carbohydrate stores in spore germination are well-described, their contributions to sustaining hyphal growth or sporulation is not understood [68, 69]. Oomycete hyphae are coenocytic and contain large vacuoles, especially in older cultures. In plants and yeast, the vacuole is an important store of nutrients [70, 71], and whether the oomycete organelle plays a similar role is unknown.

## Materials and methods

### Identification and annotation of metabolic genes

Predicted proteins from *Ph*. *infestans* strain T30-4 were used as inputs for the KEGG-based KAAS and BlastKoala annotation servers [20, 21], which predicted functions and assigned proteins to KEGG orthology (KO) groups. Some metabolic genes were also identified using GO terms associated with each gene. The proteins were also used as queries in BLASTP (2.2.31+) searches against the NR database using a cut-off *E*-value of 1e-20, and the Conserved Domain Database (CDD) using a cut-off *E*-value of 1e-10. If the KEGG and BLAST-derived annotations were in conflict, we relied on the BLAST result only if supported by the CDD results. Pathway information was obtained using data from KEGG, supplemented with information from Metacyc [72] and eggNOG [73]. When grouping enzymes into pathways for expression analysis, the KEGG classification was used except that separate pathways were made for genes involved in the metabolism of inorganic nitrate and sulfate. Enzyme Commission (EC) numbers were obtained using the Expasy database. In some cases, enzymes could not be assigned to a specific EC number and were classified as a general oxidoreductase, dehydrogenase, etc.

Genes from *Py*. *ultimum* were annotated essentially as described above. In addition, orthologs between the two species were identified using OrthoMCL, and discrepancies in annotation were evaluated manually. If a protein from one species lacked orthologs in the other, a search was made of the genome assembly to identify cases where a gene was present but had failed to be annotated. In addition to searching the T30-4 assembly of *Ph*. *infestans* released in 2009 [74], we also examined a new assembly of strain 1306 based on long-read data and optical mapping [75].

After we completed our annotations of *Ph*. *infestans*, Rodenberg et al. [76] reported identifying 1,301 genes by matching *Ph*. *infestans* sequences to custom hidden Markov models of metabolic proteins from other organisms. All proteins from our analysis were present within the list generated by Rodenberg et al. A second group [77] reported identifying 1,375 genes using the KAAS tool, but these could not be compared directly to ours since their publication did not include the gene identifiers.

Analyses of sequences from other oomycetes were performed using the Fungidb.org and EuMicrobeDb.org databases [22, 23]. The sequence of NR from *Ph*. *mirabilis* were assembled from whole-genome sequencing data deposited in Genbank.

### Growth of pathogens

Isolate MS-1 of *Py*. *ultimum* var. *ultimum* was provided by Dr. M. Stanghellini and isolated from potato in Riverside County, California. It was maintained at 21°C in the dark on half-strength V8 medium containing 1.5% agar. Strains of *Ph*. *infestans* and *Ph*. *mirabilis* were maintained in the dark at 18°C on rye-sucrose agar [78]. These were isolate 1306 collected from tomato in San Diego County, California (A1 mating type, pathogenic on tomato and potato) and others provided by Drs. W. Fry and N. Grünwald including genotypes US-11 (A1, pathogenic on both tomato and potato), US-22 (A2, more pathogenic on tomato than potato), US-23 (A1, more pathogenic on tomato than potato), and US-24 (A1, much more pathogenic on potato). Strains P19917 and P19930 of *P*. *mirabilis* (A2 and A1 mating types, from Mexico) were purchased from a culture collection. The identity of these species was confirmed by sequence analysis of their untranscribed spacer regions. Besides growth rate measurements which used all isolates, other studies used strains MS-1, 1306, and P19917.

Growth curves were performed using the media described by Henninger with 10 mM nitrate or ammonium substituted for amino acids [43]. Cultures for gene expression studies were grown using rye broth, pea broth, or infected tubers. The latter used cultivar Russet Burbank as described [19]. In brief, tubers were cut into 2 mm slices using a mandoline, and placed in a clear plastic box on a metal rack above moist paper towels. Infections were performed with three biological replicates (triplicates), each executed in separate weeks. For inoculations with *Ph*. *infestans*, 10^5^ zoospores were spread on the upper surface of each tuber slice using a rubber policeman. Slices were kept at 18°C in the dark and frozen in liquid nitrogen after 1.5 and 4 days. Sporulation began on day 3. Minor darkening of the host tissue was observed under the sporangia-bearing aerial hyphae. Since *Py*. *ultimum* does not produce zoospores, infections were performed by placing a 2-mm plug from the growing edge of a 2-day agar culture in the center of each tuber slice. After 1.5 days, tuber tissue was dissected into concentric rings representing early and late stages of colonization. The outer (early infection) ring included a 3-mm region comprised of one-quarter of nondiscolored tuber tissue and three-quarters reddish brown tissue. The inner (late infection) ring included 3-mm of black tissue.

Other defined media tested included those described by Hohl [42], Xu [79] and Scheepens et al. [80]. Broth cultures used for mRNA, protein, or metabolite studies used 100 or 150-mm diameter plates with 3.1 μl of media per mm^2^.

### Expression analysis of single genes and pathways

RNA was obtained from the strains described above by grinding tissue to a powder under liquid nitrogen, followed by extraction using Sigma Plant RNA kits. RNA-seq was performed as described and the data are available at NCBI GEO under Bioproject PRJNA407960 [19]. In brief, after quality control indexed libraries were prepared using the Illumina Truseq kit v2. Paired-end libraries were quantitated by Qubit analysis, multiplexed and sequenced using Illumina technology. Reads passing the quality filter were aligned to each pathogen’s genome using Bowtie 2.2.5 and Tophat 2.0.14, allowing for one mismatch. This used the reference genomes of *Ph*. *infestans* isolate T30-4 and *Py*. *ultimum* isolate DAOM BR144 [74, 81]. Expression and differential expression calls were made with edgeR using TMM normalization, a generalized linear model, and FDR calculations based on the Benjamini-Hochberg method [82]. However, most comparisons in this paper were performed using FPKM values to minimize artifacts resulting from errors in gene models.

For studying the aggregate expression of pathways, genes were grouped by metabolic pathway according to the assignments in S1 Table, and then their FPKM values were added together. Many enzymes were represented in multiple pathways. Genes predicted to encode proteins with very broad activities, such as genes annotated unclassifiable oxidoreductases/dehydrogenases, were generally excluded from the analysis.

RT-qPCR was performed as described [19]. In brief, primers (S2 Table) were designed to amplify bands from the 3′ end of genes. cDNA made using the Maximal First-Strand RT-PCR Kit were analyzed using a CFX Connect System (Biorad) using Dynamo HS SYBR Green qPCR kit (Thermo Scientific). Assays were based on a minimum of two biological replicates using three technical replicates per tissue sample. Control amplifications were performed using no reverse transcriptase, and melt curves confirmed the fidelity of the amplification. Gene expression was normalized using the gene for ribosomal protein S3A.

Expression data from *Ph*. *capsici* were obtained from ArrayExpress accession E-MTAB-5620. Reported in Results are the median-normalized expression values from 8 and 24 hr dpi, based on three biological replicates [48]. Expression of the *Hmp1*, *Npp1*, and *Cdc14* marker genes indicated that these timepoints represent biotrophic and necrotrophic stages similar to the early and late tuber timepoints described for *Ph*. *infestans*.

### Metabolite analysis

For calculations of nitrate and ammonium from plant samples, materials were weighed, lyophilized, ground into a fine powder using an electric mill, and provided to the Analytical Lab of the University of California, Davis for analysis. Nitrate and ammonium were assayed using a diffusion-conductivity method followed by conductivity detection [83]. Free amino acids in tubers were analyzed in-house from similar materials using a ninhydrin assay method against extracts made using 10 mM HCl and with glycine as a standard, after eliminating proteins by sodium tungstate precipitation [84].

To determine ammonium and amino acid levels in spent media, broth was separated from hyphae by filtration, frozen, and provided to the Molecular Structure Facility at the University of California, Davis for analysis. Samples were acidified with sulfosalicyclic acid to remove protein prior to analysis using a Hitachi L-8900. Samples were spiked with aminoethylated cysteine to allow quantification.

Lipid determinations were carried out using a solvent extraction method [85]. This involved harvesting hyphae by vacuum filtration on Whatman type 54 paper, and then adding to 1.5 g of ground tissue 10 ml of chloroform plus 5 ml of methanol. After shaking at 125 rpm for 30 min, 4 ml of 0.73% NaCl was added to produce a 2:1:0.8 mixture of chloroform:methanol:water (v/v/v). After further shaking, the mixture was passed through a Whatman GF/C filter and phases were separated by centrifugation at 350 ×*g* for 3 min. The bottom organic layer was then transferred to an aluminum planchet, dried, and weighed on a vintage Mettler B6 analytical balance.

Untargeted metabolomics was performed using mycelia harvested from rye media broth cultures at the timepoints described in Results. Hyphae were rinsed in water to remove media and harvested by vacuum filtration. After weighing the samples, they were frozen in liquid nitrogen and provided to the West Coast Metabolomics Center at the University of California-Davis for analysis. This involved separation by gas chromatography followed by analysis by mass spectrometry. Compounds were identified using in-house spectra and standards from Massbank of North America. Isotopic enrichment analysis was performed by spiking the broth media with 10 mM of the ^15^N compounds described in the text, using six biological replicates, and harvesting the samples at the "early" and "late" timepoints as defined in the text. Data analyses determined relative levels of each metabolite and the ^15^N/^14^N percent atom enrichment. The results may be approximate due to kinetic isotope effects [86].

### Nitrate reductase assays

Hyphae were grown in clarified rye media with or without 10 mM nitrate. For assays of enzyme activity, cultures were harvested at the early and late timepoints by vacuum filtration and rinse with water to remove nitrate. Tissue was ground under liquid nitrogen and the resulting powder mixed with extraction buffer (20 mM Tris-HCl pH 8.0, 150 mM NaCl, 10 mM EDTA, 0.2% NP-40, 0.02 mg/ml Heparin, 1mM PMSF, 1.5 mM DTT). After mixing on ice for 30 min, the mixture was centrifuged at 5000 ×*g* for 15 min at 4°C, and the supernatant was used for enzyme assays. A direct nitrate assay based on a protocol from Sigma Aldrich was then used. Assays were performed in 1 ml of 53 mM potassium phosphate buffer pH 7.5, 5 μM flavine adenine dinucleotide (FAD), 0.2 mM β-nicotinamide adenine dinucleotide phosphate (β-NADPH), and 10 mM potassium nitrate solution (or water for blanks). Protein extract (20, 20, and 40 μl for *Ph*. *infestans*, *Py*. *ultimum*, and *Ph*. *mirabilis*, respectively), typically containing 100 to 200 μg of protein, was added to start the reaction and the oxidation of NADPH at room temperature was followed at 340 nm, with timepoints taken at approximately 15 minute intervals over 2 hr. Assays were performed at 21°C, except as indicated in Results. All were done with three independent biological replicates. Protein concentrations were determined using a BCA assay kit (Pierce).

Determinations of K_m_ were performed with late timepoint samples grown with 10 mM nitrate using nine concentrations of nitrate (2-fold dilutions) ranging from 0 to 8000 μM. A graph of the rate of reaction (μmole min^-1^) versus nitrate concentration (μM) was plotted and K_m_ estimated by non-linear regression analysis [87]. These determinations used extracts from the oomycetes and enzyme from *A*. *niger* from Sigma-Aldrich.

### Immunoblot analysis

Polyclonal antibodies against NR were obtained from Agrisera (catalog number AS08 310). Blotting was performed using HRP-labeled anti-rabbit IgG as described [88]. As a positive control, the *Ph*. *infestans* gene was expressed by cloning a PCR-amplified fragment into the *Bgl*II/*Hin*dIII sites of pBAD/HISB (Invitrogen). Protein was prepared from arabinose-induced TOP10 *E*. *coli* cells, which were boiled in sample buffer prior to electrophoresis. As a negative control, protein was obtained from two transformants silenced for the *Ph*. *infestans* NR gene [47].

## Supporting information

S1 Fig Comparison of expression of metabolic genes during tuber infection and on artificial media. (PDF)

S2 Fig Infection symptoms and tissue sampling scheme. (PDF)

S3 Fig Immunoblot detection of nitrate reductase protein from *Ph. mirabilis*. (PDF)

S1 Table Gene annotations. (XLSX)

S2 Table Oligonucleotides used in polymerase chain reaction. (DOCX)
