# Supplementary material for: Niche-specific metabolic adaptation in biotrophic and necrotrophic oomycetes is manifested in differential use of nutrients, variation in gene content, and enzyme evolution
Source: PLoS Pathog. 2019 Apr 19;15(4):e1007729. doi: 10.1371/journal.ppat.1007729 (PMC6493774; doi:10.1371/journal.ppat.1007729)

S1 Figure

Comparison of expression of metabolic genes during tuber infection and on artificial media. The data are formatted as in Fig 2, except that comparisons of additional samples are included including pea media and late timepoints. When interpreting results from late samples, it should be noted that *Ph. infestans* is a profuse sporulator compared to *Py. ultimum,* and that mRNAs for many metabolic pathways decline during sporulation.

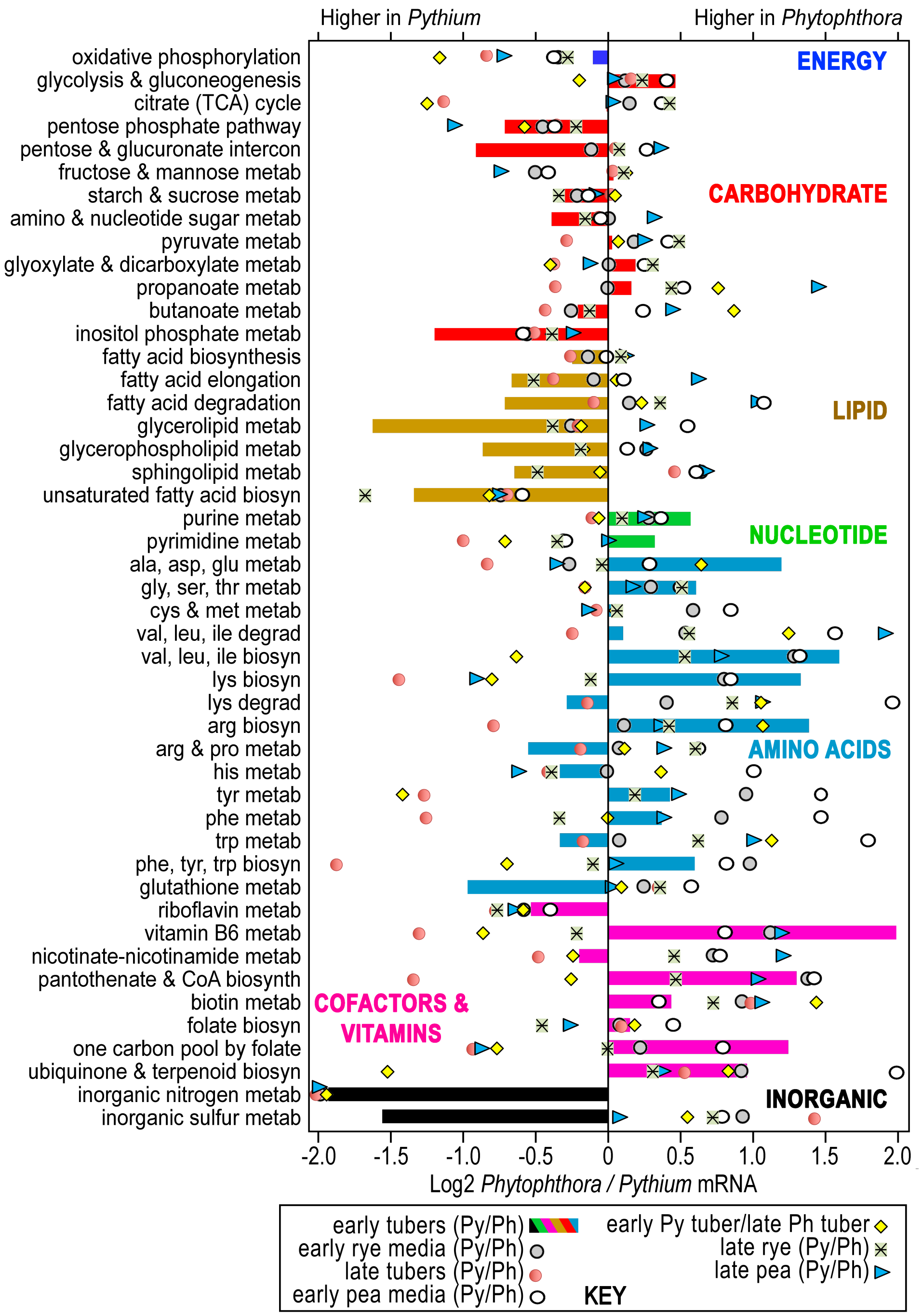

Supplement: S1 Fig — (PDF) [file ppat.1007729.s001.pdf]
