# Supplementary material for: Niche-specific metabolic adaptation in biotrophic and necrotrophic oomycetes is manifested in differential use of nutrients, variation in gene content, and enzyme evolution
Source: PLoS Pathog. 2019 Apr 19;15(4):e1007729. doi: 10.1371/journal.ppat.1007729 (PMC6493774; doi:10.1371/journal.ppat.1007729)
Supplement: S2 Fig — (PDF) [file ppat.1007729.s002.pdf]

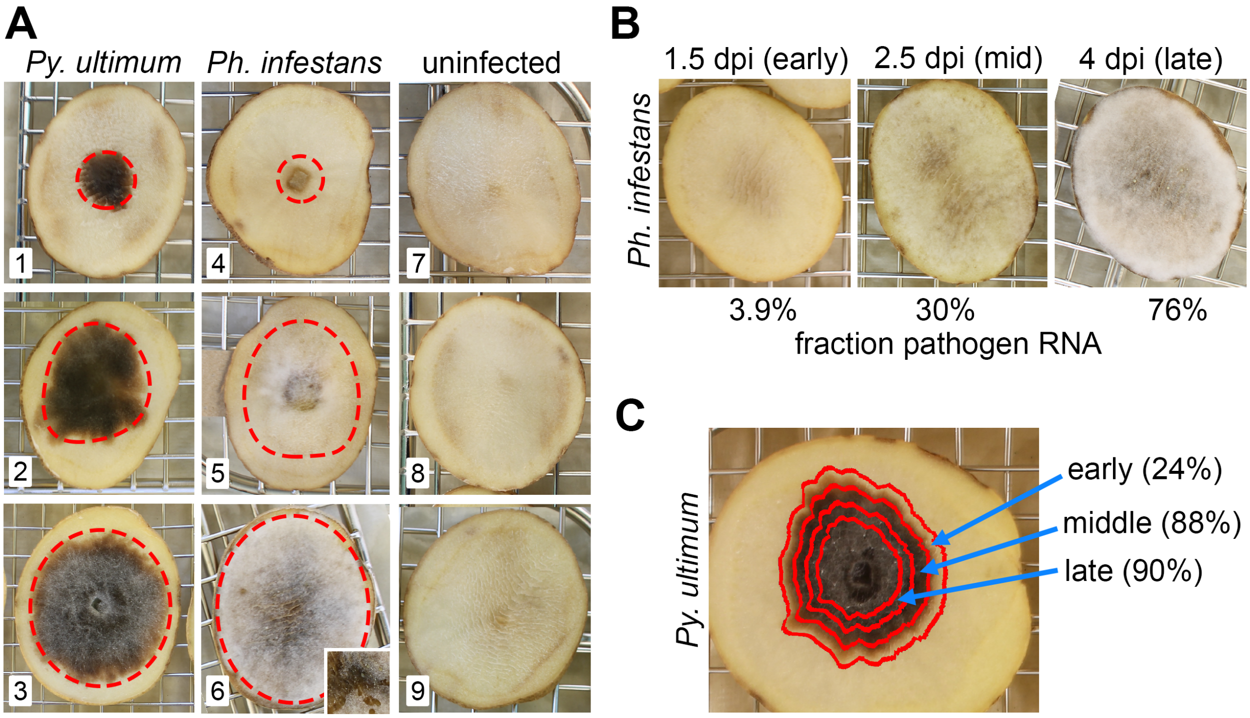

S2 Figure. Infection symptoms and tissue sampling scheme.
(A) Images of tuber slices infected with *Py. ultimum* over a 4 day time-course, and *Ph. infestans* over a 6 day time-course. Images are representative of the early (1, 4), middle (2, 5), and late stages of colonization (3,6). Also shown are mock-infected controls (7, 8, 9). Dotted red lines represent approximate boundaries of the pathogen based on microscopic analysis. For *Py. ultimum*, strong host necrosis was observed at each stage, with the host tissue becoming blackened, soft, and watery. For *Ph. infestans,* the infected tissue remained firm at each timepoint. In panel 5, *Ph. infestans* has grown across most of the tuber and is sporulating; the white material on the surface of the tuber are hyphae and sporangia. Host necrosis is only evident near the inoculum plug. Panel 6 shows *Ph. infestans* sporulating over the entire tuber surface, with necrotic tissue underlying the hyphal mat; the inset shows darkened tuber tissue in a region where the hyphae were scraped off.
(B) Sampling scheme for RNA-seq analysis of *Ph. infestans.* Zoospores were spread across the tubers, and tissues were harvested at the indicated timepoints. Shown below the images are the fraction of RNA-seq reads mapping to the pathogen genome.
(C) Sampling scheme for RNA-seq analysis of *Py. ultimum.* Tubers were inoculated with a plug of the pathogen. After 1.5 days, 3-mm rings were excised which corresponded to early, middle, and late stages of colonization. Indicated to the right of the image are the percentages of RNA mapping to the pathogen genome from each ring.
