# Supplementary material for: Niche-specific metabolic adaptation in biotrophic and necrotrophic oomycetes is manifested in differential use of nutrients, variation in gene content, and enzyme evolution
Source: PLoS Pathog. 2019 Apr 19;15(4):e1007729. doi: 10.1371/journal.ppat.1007729 (PMC6493774; doi:10.1371/journal.ppat.1007729)

S3 Figure. Immunoblot detection of nitrate reductase protein from *Ph. mirabilis.* The experiment is the same as in Fig. 9D, but with new protein extracts. The tissue samples were taken from early (E) or late (L) timepoints in rye broth media supplemented with 10 mM potassium nitrate (+) or unsupplemented media (-). The top panel shows a western blot using anti-nitrate reductase with hyphae from early and late timepoint cultures, grown with and without 10 mM potassium nitrate. The lower panel is a Ponceau-stained membrane. The arrow indicates the position of the nitrate reductase protein.

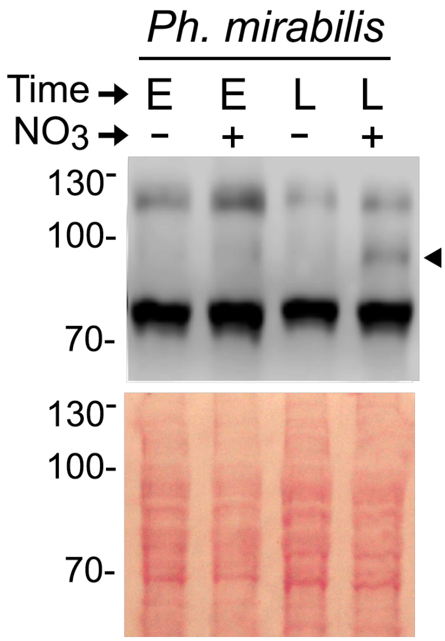

Supplement: S3 Fig — (PDF) [file ppat.1007729.s003.pdf]
