# Supplementary material for: Niche-specific metabolic adaptation in biotrophic and necrotrophic oomycetes is manifested in differential use of nutrients, variation in gene content, and enzyme evolution
Source: PLoS Pathog. 2019 Apr 19;15(4):e1007729. doi: 10.1371/journal.ppat.1007729 (PMC6493774; doi:10.1371/journal.ppat.1007729)
Supplement: S2 Table — (DOCX) [file ppat.1007729.s005.docx]

**S2 Table**

**Oligonucleotides used in PCR**

| Description | Sequence (5' to 3') | Application |
| --- | --- | --- |
| *Ph. infestans* NR | CGTACTACGGAGACGGCAATTT, CCATGGCAAAGTTAAGTCGACTCTTA | RT-qPCR |
| *Ph. infestans* NIR | GTCACCTGAACTCCTGGCTCAGTT, GGACACATGTTCTGGGTGGCATA | RT-qPCR |
| *Ph. infestans* NRT | GGCCGAATCGGAGTGCAGTTTAT, GCTGCGGCAACAAAGATACTCAA | RT-qPCR |
| *Ph. infestans* RPS3A | TAAGACGACGGACGGATAC, AGGCACAAACTTCAGGAATAG | RT-qPCR control |
| *Ph. infestans* NR | CAGAGATCTATGGCGCAAATTGACCCCAGAGAC, CGGAAGCTTAAAACACGGTGGTCTGGGTTTTGAG | Protein expression |
| *Py. ultimum* NR | CAGAAACTGAAGCCAGCAAAC, GGTGTCGAGGTCGTAGTGAAG | RT-qPCR |
| *Py. ultimum* NiR | CGAAAGAAACCTGCATCCTTGAGCTA, GGATGCCCTGCGCCAAGACGAAT | RT-qPCR |
| *Py. ultimum* NRT | GTCGGTTCCAGTCGTTGT, GAGGAAGCCCCAGCAGAG | RT-qPCR |
| *Py. ultimum* RPS3A | TGCCATCCGTAAGAAGATGAC, CTTTGGCTTCTTGAGGACCTT | RT-qPCR control |
| *Ph. mirabilis* NR | CGTACTACGGAGACGGCAATTT, CCATGGCAAAGTTAAGTCGACTCTTA | RT-qPCR |
| *Ph. mirabilis* NIR | GCAGCTTCGTCGTGTAGT, CGGTCTTCATCTGCACTT | RT-qPCR |
| *Ph. mirabilis* NRT | CGCTTTGTGTTGGAGTTT, ACCGAGTCATCTTTCTCC | RT-qPCR |
| *Ph. mirabilis* RPS3A | TAAGACGACGGACGGATAC, AGGCACAAACTTCAGGAATAG | RT-qPCR control |
